# Multifunctional implantable hydrogels: Smart platforms at the forefront of biomedical innovation

**DOI:** 10.1016/j.mtbio.2026.102940

**Published:** 2026-02-16

**Authors:** Bruna E. Nagay, Leila Mamizadeh Janghour, Labiba K. El-Khordagui, Behnam Akhavan, Valentim A.R. Barão, Vimukthi Dananjaya, Chamil Abeykoon, Salma E. El-Habashy, Jagan Mohan Dodda

**Affiliations:** a Department of Prosthodontics and Periodontology, Piracicaba Dental School, Universidade Estadual de Campinas (UNICAMP), Piracicaba, São Paulo, 13414-903, Brazil; b School of Biomedical Engineering, Faculty of Engineering, University of Sydney, Sydney, NSW, 2006, Australia; c Department of Pharmaceutics, Faculty of Pharmacy, Alexandria University, Egypt; d School of Engineering, College of Engineering, Science and Environment, University of Newcastle, Callaghan, NSW, 2308, Australia; e Hunter Medical Research Institute (HMRI), Precision Medicine Program, New Lambton Heights, NSW, 2305, Australia; f Sydney Nano Institute, University of Sydney, Sydney, NSW, 2006, Australia; g School of Physics, Faculty of Science, University of Sydney, Sydney, NSW, 2006, Australia; h School of Engineering, Swinburne University of Technology, Hawthorn, VIC, 3122, Australia; i Northwest Composites Centre and Henry Royce Institute, Department of Materials, Faculty of Science and Engineering, The University of Manchester, Oxford Road, Manchester, M13 9PL, UK; j New Technologies – Research Centre (NTC), University of West Bohemia, Univerzitní 8, Pilsen, 301 00, Czech Republic

**Keywords:** Additive manufacturing, Hydrogel ink formulation, Injectable biomaterials, Antimicrobial hydrogels, Bone tissue engineering, Real-time health monitoring, Personalized medicine

## Abstract

Hydrogels are transformative three-dimensional polymeric networks that replicate the extracellular matrix owing to their high-water content, biocompatibility, and tunable physicochemical properties. Evolving beyond conventional applications in wound dressings, contact lenses, and basic drug depots, hydrogel systems have advanced into implantable designs capable of long-term physiological integration. Surgically placed or delivered via minimally invasive techniques, implantable hydrogels (IHGs) enable dynamic tissue interactions, biodegradability, self-healing behaviour, and sustained drug release. The emergence of multifunctional, stimuli-responsive variants of IHGs has further expanded their therapeutic, diagnostic, and regenerative potential while preserving their essential material attributes. By coupling stimuli responsiveness with patient-specific physiological cues, IHGs embody the “smart” nature of next-generation biomaterials, advancing personalized medicine through adaptive therapeutic delivery, real-time functional responsiveness, and dynamic biological integration. This review summarizes recent progress in the design and fabrication of IHGs, emphasizing 3D and 4D printing technologies and the development of hydrogel inks optimized for mechanical robustness, shape fidelity, and biological performance. Applications are discussed across four major areas: (i) hydrogel coatings for medical implants, (ii) injectable hydrogels for infection control, (iii) bone-regenerative scaffolds, and (iv) health-monitoring systems. Finally, the review addresses key translational challenges, including scalable manufacturing, long-term stability, and regulatory considerations, while outlining future directions toward smart, multifunctional implantable hydrogels capable of integrated biosensing and responsive therapeutic delivery. Distinct from previous reviews, this work combines implantability and multifunctionality/smartness within a single framework, highlighting how hydrogels can achieve durable physiological integration while dynamically adapting to patient-specific cues.

## Graphical abstract

**Figure:**
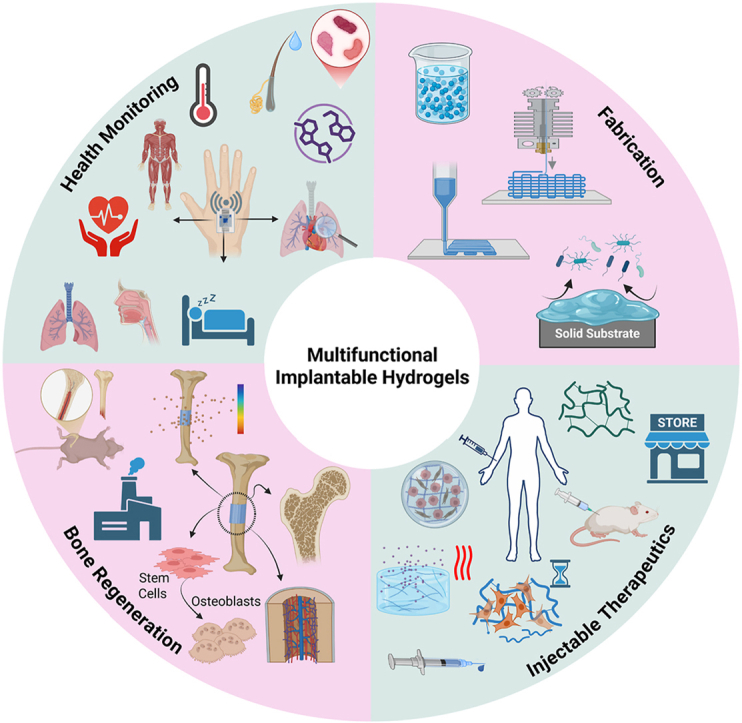

## 1 Introduction

Hydrogels, defined as 3D crosslinked polymeric networks, exhibit biocompatibility, high water absorption capacity, and the ability to mimic the extracellular matrix (ECM) while encapsulating and delivering cells and therapeutics [1,2]. These characteristics have positioned hydrogels as a pivotal platform in biomedical science and clinical applications. Continued advances in materials science, fabrication technologies, and biological understanding have transformed conventional hydrogels into sophisticated multifunctional biomaterials with adaptive properties and customizable architectures. As next-generation biomaterials, hydrogels are demonstrating significant clinical relevance across a broad range of controlled drug delivery, as well as therapeutic, regenerative and diagnostic applications [[3], [4], [5], [6]].

Among the various hydrogel platforms, implantable hydrogels (IHGs) designed for surgical or minimally invasive placement into the body, have attracted considerable interest for their potential application as localized therapeutic platforms [7], regenerative scaffolds [8], wearable health monitoring devices [4], and human-machine bio-interfaces [1,9,10]. IHGs can be broadly classified based on their physical state and mode of administration into solid preformed matrices [11] and injectable, in situ-forming liquid systems [12]. Preformed IHGs are synthesized and crosslinked ex vivo, allowing immediate function upon implantation [13,14]. They offer high shape fidelity, mechanical stability, and structural precision, making them suitable for bone and cartilage regeneration [6,15], wound healing [16], ocular implants [17] and sustained drug delivery systems [11]. However, implantation often requires invasive procedures, especially for non-biodegradable systems, posing risks in sensitive areas such as the eye [18]. Their fixed geometry also limits adaptability to irregular defects, reducing utility in complex tissue environments [19]. On the other hand, injectable in situ-forming HGs are delivered as liquid precursors that gel under physiological conditions, forming ECM-like 3D networks [20]. Gelation can be triggered by temperature, pH, enzymes, light, or ionic interactions, and is tailored to specific applications [21]. Some injectable hydrogels are shear-thinning preformed types that regain their structure after injection [22]. Injectable HGs allow minimally invasive delivery conforming to complex anatomies, enhancing tissue integration. Their stimuli-responsive nature supports controlled release of drugs, biotherapeutics, cells, and nanoparticles [[23], [24], [25], [26]]. However, they generally have lower mechanical strength post-gelation compared to their preformed counterparts and face challenges in gelation consistency, reproducibility, and scalable manufacturing [27].

Multifunctional IHGs have emerged as the next-generation systems representing a transformative class of biomaterials designed to integrate multiple capabilities, such as stimuli-responsiveness, controlled therapeutic delivery, and real-time monitoring, within a single platform. This multifunctionality significantly enhances therapeutic efficacy and expands their potential across diverse clinical settings [28,29]. Thus, to meet the complex demands of biomedical applications, multifunctional IHGs are engineered with precisely tailored material properties [30]. A critical attribute is mechanical tunability, which ensures that the hydrogel's stiffness, elasticity, and toughness align with the mechanical characteristics of target tissues [30]. This tunability is achieved by varying the polymer type/concentration [31], crosslinking density/method [32] as well as the addition of fillers such as nanoparticles [33], nanocrystals [34] and nanofibers [35]. Beyond mechanical adaptation, many IHGs are designed to possess self-healing and stretchable properties, through dynamic bonding strategies. These features enable the hydrogel to withstand physiological movements and recover from damage, a property of importance for strain/pressure wearable devices [36] and anti-freezing hydrogels [37,38]. Stimuli-responsiveness is another hallmark of advanced IHGs. Sensitivity to temperature, pH or redox conditions [39,40] enables on-demand functionalities for applications such as controlled drug release [41], wound healing [42], and endodontics [43]. In parallel, the incorporation of electrically conductive components, such as polypyrrole or graphene, allows the hydrogels to interface with various tissues for wearable sensing [44] and to promote the healing of chronic wounds [45,46]. Equally important, biocompatibility is ensured by using natural polymers, often combined with bioactive cues for therapeutic and tissue regeneration applications [47]. For temporary implants, controlled degradability is achieved using biodegradable polymers, eliminating the need for surgical removal [48,49]. Finally, strong tissue adhesion, often inspired by mussel-inspired catechol chemistries, has been reported to facilitate intimate integration with host tissues, further improving clinical performance [50].

Altogether, these properties position multifunctional IHGs as versatile platforms for diverse applications. For example, in therapeutic delivery, their stimuli-responsiveness and degradability can enable controlled release of drugs or bioactive agents, enhancing treatment efficacy while minimizing systemic side effects. This is particularly of benefit in cancer therapy [51,52], chronic inflammation [53], and infection control [54]. As tissue-supporting scaffolds, their mechanical tunability, biocompatibility, and bioactivity promote cell adhesion and regeneration, supporting repair in bone and cartilage [6], spinal cord [49], and cardiac tissues [55]. In bioelectronics, their conductivity and compliance with soft tissues enable real-time sensing and stimulation, advancing applications such as implantable biosensors [56], electroactive wound dressings [46], and neural interfaces [57,58].

**Fig. 1:**
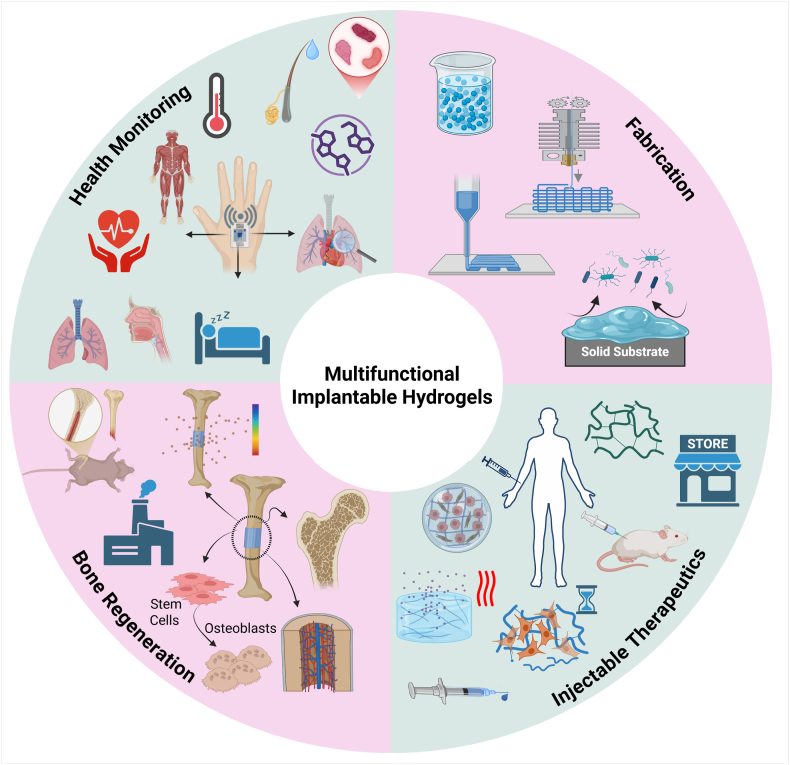
Schematic overview of multifunctional implantable hydrogels (IHGs) and their main application domains. The central circle highlights the integrative role of IHGs in biomedical applications, while the surrounding segments illustrate key areas covered in this review: additive manufacturing strategies for hydrogel fabrication (top right), including 3D printing and tailored ink formulations; hydrogel coatings for implants via covalent and non-covalent attachment methods; injectable hydrogels for infection control, addressing design, synthesis, and translational aspects; IHGs engineered for bone defect repair with emphasis on osteoconductivity, immunomodulation, and vascularization; and hydrogels designed for real-time health monitoring, including biochemical, physiological, and disease-related parameters. Created with Biorender.com.

Thus, herein, we consolidate core design principles with a critical appraisal of translational challenges, providing a comprehensive overview of multifunctional IHGs. Special emphasis is given to fabrication and design strategies—particularly those enabled by advanced 3D and 4D printing technologies—and their applications in four primary domains: (i) hydrogel coatings for implants via covalent and noncovalent attachment; (ii) injectable hydrogels for infection control; (iii) scaffolds for bone regeneration tailored to biological performance requirements; and (iv) hydrogels for health monitoring systems (Fig. 1). This work distinguishes itself from earlier literature by offering an integrative perspective spanning antimicrobial coatings, injectable formulations, bone-regenerative scaffolds, and biosensing platforms. It highlights advances in additive manufacturing, covalent and noncovalent surface modification, and stimuli-responsive drug delivery. Regulatory considerations, preclinical evaluation models, and key translational barriers are also discussed.

## 2 Additive manufacturing of implantable hydrogels

The fabrication of implantable hydrogels through additive manufacturing technologies including 3D and 4D printing has made it possible to make highly precise, patient-specific clinical devices for drug delivery and tissue engineering [59]. Recent work documented the utilization of hybrid hydrogels composed of natural polymers such as alginate [60], gelatin [61], and hyaluronic acid [62] with their synthetic counterparts, i.e., polyethylene glycol (PEG) and polyvinyl alcohol (PVA), to obtain superior mechanical properties (compressive strength >50 kPa) and regulated degradation rates to match the implant condition [63]. Advances in 3D printing technologies, such as micro extrusion and stereolithography, enable accurate layer-by-layer printing with 50 μm resolution, which is required for the replication of intricate tissue structure [64,65].

### 2.1 Hydrogel ink for additive manufacturing

The 3D and 4D printability of hydrogel inks largely depends on their viscosity and shear-thinning behaviour. Hydrogel inks exhibiting viscosities in the range of 0.1–10 Pa s at low shear rates facilitate smooth extrusion through printer nozzles [66]. Alginate-based inks, with a viscosity of approximately 1 Pa s at a shear rate of 1 s^−1^, demonstrate excellent extrudability and are widely regarded for their print-friendly rheological properties [67]. Shear-thinning behaviour with values of flow indices between 0.2 and 0.4 ensures smooth extrusion along with structural stability [68]. Rheological properties such as storage modulus (G′) and loss modulus (G'') play a key role in layer stacking. Inks, such as gelatin-methacrylate (GelMA), with a storage modulus greater than 1 kPa— typically ranging from 1 to 5 kPa—demonstrate excellent shape retention [69]. Rapid gelation kinetics, with a crosslink time of less than 30 seconds, also lend extra structural strength in extrusion-based printing, while low-viscosity inks offer resolutions less than 100 μm in stereolithography [70].

Hydrogel-based ink formulations are designed to address the requirements of specific 3D and 4D printing processes. For extrusion printing, high shear recovery (viscosity recovery >90% in 5 seconds) ensures continuity, and inks containing 3–5 wt % alginate achieve resolutions of around 200 μm for vascular networks [71]. Vat photopolymerization inks typically need low viscosities between 0.01 and 0.1 Pa s and photoinitiator concentrations of 0.05–0.1 wt%, enabling fast polymerization and the formation of fine microstructures with layer thicknesses of 50–100 μm [72]. Stimuli-sensitive hydrogels such as PNIPAM enable high-performance 4D printing by achieving programmed deformations with expansion ratios above 300% and activation times under 5 minutes. This rapid, large-scale response allows the creation of dynamic structures for biomedical uses, including drug delivery systems and tissue scaffolds [73].

The network architecture and crosslinking density established during printing govern degradation rates, swelling behavior, and nutrient diffusion, which are essential for supporting cell viability, tissue ingrowth, and controlled release of bioactive molecules [74,75]. Moreover, the microstructural precision achievable via extrusion or vat photopolymerization printing can be leveraged to create porous networks that enhance vascularization and tissue integration, a critical requirement for implantable scaffolds [76]**.** Thus, the careful tuning of printability parameters translates directly into constructs that meet the mechanical, biological, and functional demands of implantable hydrogel systems.

Building on these considerations, the functional performance of implantable hydrogels can be further enhanced by combining multiple materials or bioactive components within a single construct. Multimaterial strategies allow designers to spatially tune mechanical properties, degradation profiles, and biological cues, thereby extending the capabilities of 3D and 4D printed hydrogels beyond what is achievable with a single ink formulation. In such systems, “multimaterial” refers to the integration of two or more hydrogel formulations and/or functional constituents (e.g., polymers with distinct rheological or responsive properties, reinforcing or conductive particles, and living cells) within a single printed construct, often featuring tailored, functional, or gradient properties to achieve spatially heterogeneous composition and functionality. Multimaterial printing enables, for example, the simultaneous printing of different colors, varied mechanical properties like stiffness, or embedded electronics [77]. This shift from single-ink optimization to coordinated ink integration necessitates compatibility in rheological windows, interfacial adhesion, and crosslinking kinetics, enabling seamless material transitions and the fabrication of hierarchically functional hydrogel architectures.

**Fig. 2:**
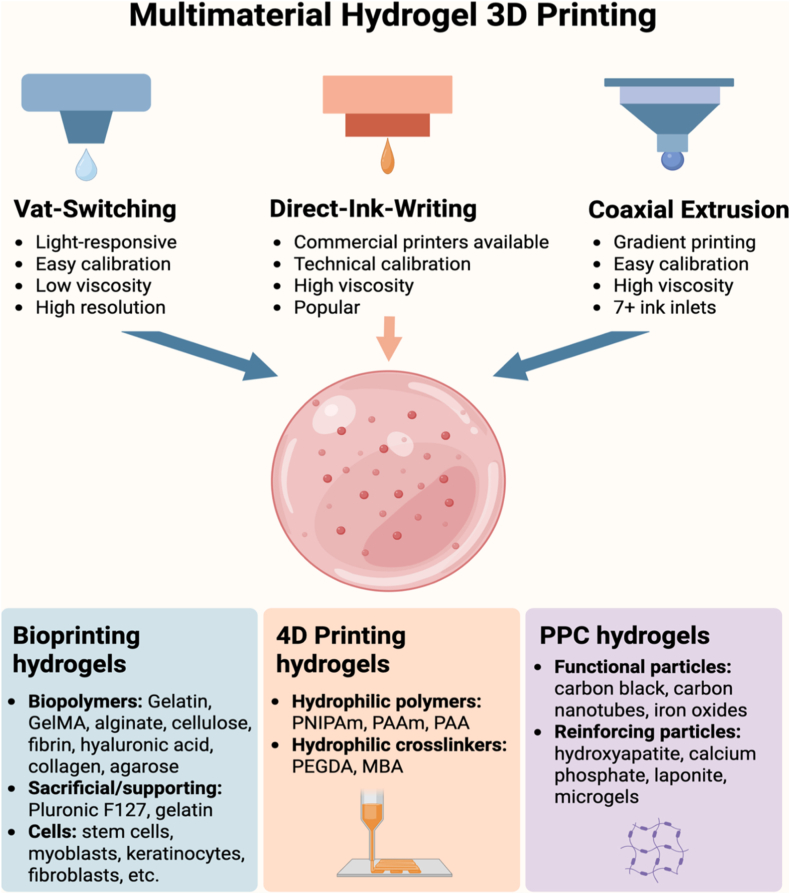
Multimaterial hydrogel additive manufacturing, defined here as the integration of two or more hydrogel inks with distinct chemical compositions, physical properties, or biological functions within a single printed construct. Vat-switching enables multimaterial fabrication in light-based printing (SLA/DLP) by sequentially exchanging photoinks during the printing process, allowing high spatial resolution. Direct-ink-writing (DIW) relies on extrusion of shear-thinning, viscoelastic hydrogel inks—often through multiple nozzles—to spatially pattern different materials within one structure. Coaxial extrusion delivers multiple inks through a single toolhead, enabling continuous gradients, improved interfacial alignment, and reduced geometric mismatch between materials. The lower panels illustrate typical ink components of key application domains, including bioprinting (cell-laden and biomimetic matrices), 4D printing (stimuli-responsive hydrogels capable of programmed shape or property changes), and particle–polymer composite (PPC) hydrogels incorporating functional fillers. Created with BioRender.com. Abbreviations: PNIPAm = poly(N-isopropylacrylamide); PAAm = polyacrylamide; PAA = poly(acrylic acid); PEGDA = polyethylene glycol diacrylate; MBA = N,N′-methylenebisacrylamide.

Fig. 2 presents a comprehensive summary of multimaterial hydrogel 3D printing techniques and their applications, particularly in bioprinting, 4D printing, and functional hydrogel manufacturing. It presents three dominant 3D printing techniques: Direct-Ink-Writing (DIW), Vat-Switching, and Coaxial Extrusion. DIW, a versatile and widely used technique, enables precise deposition of high-viscosity inks, making it ideal for fabricating complex 3D structures [78]. Although it benefits from technical calibration and compatibility with commercial printers, achieving a balance between ink viscosity and biocompatibility in live cell applications remains challenging [79]. On the other hand, Vat-Switching employs photo-responsive materials to achieve high resolution, making it particularly well-suited for fabricating intricate microarchitectures such as microvascular networks [80]. Coaxial extrusion stands apart for its ability to facilitate gradient printing with multiple inlets of material, which is of significant use for the fabrication of biomimetic structures, such as cartilage-bone interfaces. Future research should optimize nozzle geometries in coaxial extrusion to minimize shear stress and improve cell viability [81].

Bioprinting hydrogels rely on biopolymers, sacrificial materials, and cellular components. Common biopolymers—such as alginate, GelMA, and cellulose are favored for their biocompatibility and tunable responsiveness. However, these materials often require nanoparticle reinforcement or blending with additional polymers to enhance mechanical properties. For example, blending alginate with cellulose nanocrystals enhances stiffness and biodegradability [82]. Sacrificial substrates—such as gelatin and Pluronic F-127—provide structural support during printing but can exhibit cytotoxicity; safer alternatives include carbohydrate-based inks [83]. Incorporating diverse cell types (e.g., Schwann cells for neural tissue and cardiomyocytes for cardiac patches) highlights bioprinting's versatility [84]. However, maintaining cell viability and function both during and post-printing remains a significant challenge, and approaches such as microenvironmental conditioning during fabrication warrant further investigation.

Advances in 4D printing and PPC hydrogels continue to expand the application of this technology. 4D printing takes advantage of stimuli-responsive polymers like poly(N-isopropylacrylamide) (PNIPAm), polyacrylamide (PAAm), and poly(vinyl alcohol) (PVA), PAAS (polyacrylate sodium), and CaCl_2_) (PAA) as well as crosslinkers like poly(ethylene glycol diacrylate) (PEGDA) and N,N′-methylenebisacrylamide (MBA) like the caption of Fig. 2 to create constructs with dynamic properties like swelling or shape memory [85]. This technology opens doors to applications in soft robotics, tissue engineering, and self-folding structures. Specifically, 4D printing has revolutionized the development of smart hydrogels that can undergo time-dependent changes in shape and features in response to stimuli such as temperature, pH, humidity, ionic strength, light, and electric or magnetic fields. For example, Habib et al. achieved anisotropic 4D shape metamorphosis via digital light processing 3D printing and gradient photocrosslinking for hydrogel development for potential biomedical applications [86]. Nevertheless, ensuring long-term stability of the materials in repetitive stimuli is vital for in vivo applications. The incorporation of functional particles—such as MXenes for electrical conductivity—and reinforcing particles—like hydroxyapatite (HAp) for mechanical strength—opens up possibilities for advanced applications in bioelectronics and bone tissue engineering [87]. While these materials introduce valuable functionalities, concerns about their biocompatibility and integration remain. Addressing potential cytotoxicity and improving tissue compatibility through surface modification or the development of biodegradable alternatives will be essential for their safe translation. Collectively, these technologies represent a significant advance not only in personalized medicine but also in industrial applications, underscoring the need for multidisciplinary approaches to address challenges in scalability and standardization.

### 2.2 3D printing of implantable hydrogels

3D printing via extrusion is founded upon the precise layer-by-layer deposition of hydrogel inks to build structures. However, structural fidelity may be challenging to preserve during printing due to gravitational collapse and uncontrolled ink flow. To overcome such limitations, hydrogels with yield stress above 50 Pa have been developed to enable the ink to sustain shape under self-weight. For example, alginate-based hydrogels reinforced with 2 wt% calcium ions have demonstrated improved printability, maintaining structural fidelity in multilayer constructs up to 20 mm in height [88]. Extrusion pressures between 30 and 80 kPa and nozzle diameters between 200 and 400 μm are also optimized to minimize defect formation and facilitate uniform deposition [89].Printing parameters such as printing speed, extrusion pressure, and nozzle temperature significantly influence the quality of printed constructs. Low extrusion pressures (<30 kPa) result in incomplete or interrupted layers, while high pressures (>100 kPa) result in over-extrusion and smearing [90]. Studies have shown that printing speeds of 5–10 mm/s offer the best balance between resolution and consistency of deposition [91]. Temperature-controlled extrusion is particularly important for thermosensitive hydrogels like GelMA, where 37–40 °C nozzle temperatures are kept constant to allow free flow without pre-gelation [92,93]. These optimized parameters enable the fabrication of complex geometries, such as porous scaffolds with over 85% interconnectivity, which are crucial for effective tissue integration.

Print resolution in extrusion-based techniques is governed by nozzle diameter, rheological properties of the hydrogel, and extrusion pressure. High printing precision is particularly critical for applications such as vascular scaffolds, where pore sizes range of 200–400 μm is necessary to facilitate cell infiltration and nutrient diffusion [94]. High resolution has been facilitated by new nozzle designs, such as tapered nozzles, which have reduced filament diameters to as small as 100 μm. Moreover, shear-thinning hydrogel inks with a flow index value between 0.3 and 0.5 enable more controlled extrusion, achieving printing resolutions below 200 μm without compromising mechanical stability [95]. These advances make extrusion-based printing viable for complicated biomedical constructs. Post-printing crosslinking processes are required for enhancing the mechanical behavior and stability of hydrogel constructs without sacrificing their biological compatibility. Ionic crosslinking with divalent cations (e.g., calcium ions) has been widely employed for alginate-based hydrogels, achieving 20–30 kPa compressive strengths [96]. When compared to pure GelMA hydrogels (18–21 kPa), the hybrid hydrogel inks showed a greater compressive modulus (25–28.35 kPa) [97]. Dual crosslinking strategies, combining ionic and covalent mechanisms, further increase structural integrity, with storage moduli above 2 kPa, for long-term performance under physiological conditions [98].

Leveraging these improvements in print resolution and mechanical stability, extrusion-based techniques now support the development of hybrid composite scaffolds tailored for advanced tissue engineering. These are complex, multilayered structures designed for tissue engineering applications. They comprise a non-plastic bioink for cell incorporation and a plastic support ink to ensure mechanical strength and structural fidelity [99]. The bioink, composed of hydrogels, can facilitate cell adhesion, growth, and differentiation due to their biocompatibility and biomimicry. Conversely, the ink used for plastic support, which is typically sourced from thermoplastics such as PCL or polylactic acid (PLA), provides the mechanical integrity required to support the architecture of the scaffold on fabrication and subsequently in biological applications. The layer-by-layer arrangement allows precise spatial control over bioactive and structural components, facilitating the introduction of biomimetic gradients or zonal architectures that closely resemble native tissue. Such material integration exploits the complementary properties of each ink to enable applications in regenerative medicine, namely in load-bearing tissues or complex organs with both biological function and mechanical strength. For instance, as shown in Fig. 4, Shim et al. [100] employed PCL and chondrocyte cell-encapsulated alginate hydrogel in alternating layers to successfully 3D bioprint a multi-layered, cell-rich, and cytocompatible composite material using a multiple-head deposition system, resulting in an effective cartilage reconstruction in a murine model. The vitality of the chondrocytes was little affected by the printing process of cell-encapsulated alginate hydrogels. This technology facilitated the fabrication of distinct pre-tissue constructs by simultaneously streamlining scaffold formation and enabling the precise deposition of cells and growth factors at defined locations. Histochemical analyses conducted four weeks post-implantation revealed enhanced cartilage tissue formation and increased type II collagen fibril production within the printed PCL–alginate hybrid scaffold, with no evidence of adverse tissue reaction. Overall, multilayered 3D-printed structures demonstrate strong potential for tissue engineering by combining biological functionality with mechanical robustness.

Building on the capabilities of 3D printing to precisely engineer complex structures, 3D bioprinting extends these advantages to biological systems, enabling the spatial arrangement of living cells within tailored extracellular matrices. This approach bridges traditional tissue engineering and organoid development, providing a platform where organoids can be cultured under highly controlled conditions that mimic native tissue architecture. Organoids are advanced in vitro models in which stem cells self-organize into three-dimensional structures that mimic the architecture and function of native organs [101]**.** A key factor in organoid culture is the use of suitable scaffolds, and hydrogels have emerged as promising materials due to their biocompatibility, tunability, and degradability, which support stem cell growth and differentiation.

Combining 3D bioprinting with organoid technology offers significant advantages for tissue modeling and regenerative research [102]. Bioprinting enables the precise spatial arrangement of multiple cell types within tailored ECMs, enhancing the structural and functional fidelity of organoids. This approach improves reproducibility and scalability, allowing the production of uniform organoid arrays suitable for high-throughput studies. Furthermore, it facilitates the creation of more complex tissue models by incorporating vascular-like networks [103] and patterning the matrix microenvironment [104], better recapitulating native tissue interactions and supporting applications in drug testing, disease modeling, and regenerative medicine.

Leveraging recent advances in fabrication and design strategies, a clear classification framework for implantable hydrogels provides conceptual organization by distinguishing hydrogels according to their mode of implantation, functional integration, and application domain. IHGs can be classified by polymer source, crosslinking method, stimuli responsiveness, structure, and degradability enables precise tailoring for biomedical applications. Hydrogels are commonly divided into natural (e.g., collagen, alginate, gelatin, chitosan) and synthetic (e.g., PVA and poly(lactic-co-glycolic acid (PLGA) types) [105]. While natural polymers offer biocompatibility and bioactivity, their mechanical robustness is limited [106]. In contrast, synthetic polymers provide mechanical tunability and reproducibility, though they may require functionalization and/or or hybridization with natural polymers to enhance bioactivity [107]. Based on crosslinking, IHGs are categorized as chemically (covalently) or physically (reversibly) crosslinked. Chemically crosslinked hydrogels using crosslinking agents like genipin and glutaraldehyde, or photoinitiators to promote covalent bonding through processes like photopolymerization or enzymatic coupling, have demonstrated to offer high mechanical stability for long-term implantation [108]. In contrast, physically crosslinked hydrogels rely on reversible, non-covalent interactions, such as electrostatic interactions, hydrogen bonding, or hydrophobic associations [109]. These hydrogels are often stimuli-responsive, enabling dynamic behaviours in response to stimuli like pH, temperature, or enzymatic activity. Accordingly, IHGs may be further subclassified into responsive (smart) and non-responsive systems based on their environmental adaptability. Structurally, IHGs include single networks**,** interpenetrating polymer networks (IPNs)**,** and double-network systems**,** which enhance mechanical performance [110,111]. In terms of size, IHGs range from bulk hydrogels to microgels (10–1000 μm) and nanogels (<1000 nm), with smaller forms favored for injectability and targeted delivery [112]. Lastly, degradable IHGs—via hydrolysis or enzymatic action—are suited for transient therapies, while non-degradable variants support long-term structural roles.

## 3 Hydrogel coatings for implants

While hydrogels hold significant promise as implantable biomaterials, several challenges hinder their clinical use as standalone implants. A primary limitation is their inherent mechanical weakness, characterized by low strength and limited load-bearing capacity—which renders them unsuitable for applications such as bone or joint replacements [113]. Many implantable hydrogels also degrade over time via either hydrolysis or enzymatic reactions, which can result in a premature loss of function before fulfilling their intended purpose [114,115]. Another concern is the poor fixation and integration of the implantable hydrogels within the body, as they often lack the rigidity to anchor effectively within tissues, leading to displacement or failure over time [116]. To address these challenges, hydrogel coatings have been developed to combine the inherent biocompatibility of hydrogels with the mechanical robustness of implants by forming a solid– hydrogel platform. In such materials, the underlying implant provides the required structural integrity, while the top hydrogel coating enhances biocompatibility, cell adhesion, promotes tissue integration, and reduces immune response [[117], [118], [119], [120]]. Hydrogel coatings can also be engineered for localized and sustained delivery of drugs and biological agents at the implant site, thereby minimizing systemic side effects and further enhancing surface biocompatibility [117,118,120]. This section reviews the strategies employed for attachment of hydrogel coatings, as well as the types of hydrogel coatings utilized—particularly those aimed at preventing implant-induced infections. Current challenges associated with the application of hydrogel coatings and potential future directions in this field are also discussed.

### 3.1 Strategies for surface attachment of hydrogel coatings

#### 3.1.1 Noncovalent attachment

The non-covalent attachment of hydrogel coatings relies on intermolecular interactions between hydrogel macromolecules and the implant surface, rather than chemical bonding, leading to a reversible and dynamic surface modification. Electrostatic and hydrophobic interactions [[121], [122], [123]], hydrogen bonding [[124], [125], [126], [127]], and van der Waals forces [127] are usually involved in the non-covalent attachment of hydrogel coatings. Such methods do not require complex chemical modifications or harsh reaction conditions, preserving the structure and properties of the hydrogels. Common strategies include physical entrapment [[128], [129], [130]], adsorption-driven deposition (physisorption) [[121], [122], [123],^127^], electrostatic self-assembly [131,132], and layer-by-layer (LbL) assembly [[133], [134], [135], [136]]. This subsection explores non-covalent attachment methods and their underlying mechanisms for fabricating hydrogel coatings on the implants surface.

**Fig. 3:**
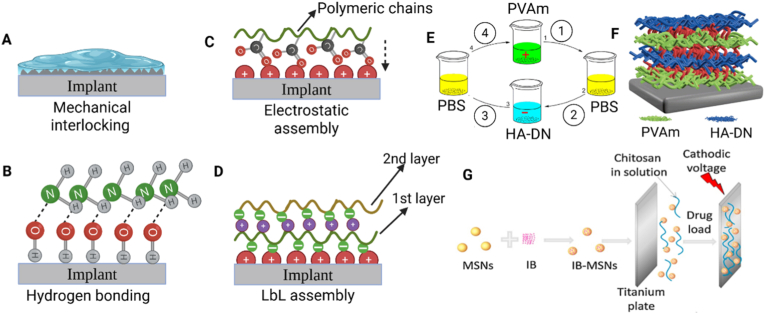
Non-covalent attachment strategies. Schematic illustrations of A) mechanical interlocking of a hydrogel coating, B) hydrogen bonding, C) electrostatic self-assembly, and D) layer by layer (LbL) assembly. All created in Biorender.com with permission. E) Fabrication steps of a coating from polyvinylamine (PVAm) and dopamine-modified hyaluronic acid (HA-DN) polymers by LbL assembly and F) schematic of the coating with successive layers of PVAm and HA-DN. Reproduced with permission [137]. Copyright 2020, Elsevier. G) Electrodeposition of a chitosan hydrogel incorporating ibuprofen (IB)-loaded mesoporous silica nanoparticles (MSNs) on a titanium plate. Reproduced with permission [138]. Copyright 2014, Elsevier.

In physical entrapment, also known as mechanical interlocking, hydrogel macromolecules are entrapped within the surface topographical features —such as micro-grooves, pores, or interwoven structures — without forming chemical bonding (Fig. 3A). This mechanism enhances coating adhesion by increasing interfacial contact area and mechanical resistance to detachment. For instance, studies using microstructured polymeric and metallic substrates, including poly(dimethylsiloxane) (PDMS) with micro-protrusions and porous titanium (Ti), have demonstrated the effects of surface structure on adhesion. These studies confirmed that greater surface roughness and porosity, strengthen hydrogel adhesion through interlocking effects [128,129]. However, since most implant surfaces lack intrinsic topographical features, additional surface pre-treatments are often required to enable effective mechanical interlocking.

**Fig. 4:**
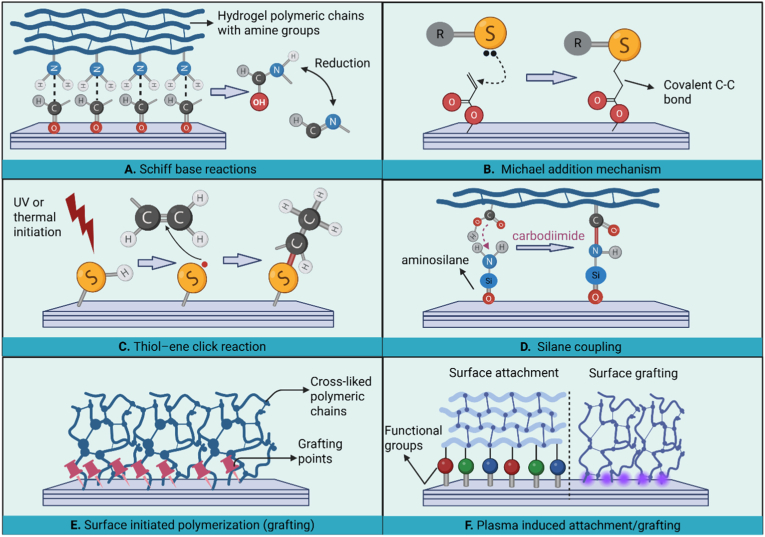
Covalent bonding strategies. Schematic illustration of A) Schiff base reactions between an aldehyde-functionalized surface and hydrogel polymeric chains with amine groups, B) Michael addition reactions between a thiol group with unpaired electrons (under oxidative conditions) as the Michael donor and an acrylate-based group on the surface, C) thiol–ene click reactions between thiol and alkene (-C=C) groups under UV or thermal initiation, D) silane coupling mechanism between an aminosilanized surface and hydrogel polymeric chains with carboxyl groups (using carbodiimide chemistry), E) surface initiated polymerization of hydrogel polymeric chains from the surface (grafting), and F) plasma-assisted surface attachment (using functional groups) and polymerization (grafting) of hydrogel coatings. Figure created in Biorender.com with permission.

Anodic oxidation, or anodization, is an electrochemical method used to create oxide layers on metallic substrates, enhancing surface roughness, porosity, and chemical reactivity [[139], [140], [141]]. These modifications strengthen the physical interlocking between hydrogel coatings and implant surfaces. For instance, a recent study on anodized Ti has shown that created nanostructures such as nanopores and nanotubes significantly improve hydrogel adhesion by increasing interfacial contact area and providing more active anchoring sites [130]. Similar surface texturing techniques such as micro-milling [142], laser processing, and laser ablation [143] can also generate hierarchical features that promote mechanical interlocking and coating stability. Physical entrapment offers a simple and effective strategy for the non-covalent immobilization of hydrogel coatings but offers limited stability under physiological conditions. Moreover, successful physical entrapment requires the implant surface to either possess inherent microtextures or be deliberately engineered to facilitate the interlocking of hydrogel chains, which further constrains the clinical applicability of this approach.

Adsorption-driven deposition, or physisorption, relies on weak intermolecular forces such as Van der Waals, hydrophobic, hydrogen, and ionic interactions to attach hydrogels to implant surfaces. Hydrophobic domains within hydrogel polymers can interact with nonpolar surface regions, facilitating the physisorption of hydrogel coatings [121]. For instance, a Janus-structured hydrogel containing hydrophobic lignin domains was applied to various substrates (e.g., glass, metal, rubber) through aggregation and surface hydrophobic interactions [121]. Hydrogen bonding of hydrogels occurs when hydrogen atoms in functional groups such as hydroxyl (-OH) or amine (-NH_2_) interact with electronegative atoms like oxygen or nitrogen on the opposing surface or within the hydrogel structure [[124], [125], [126]]. For instance, silicon hydroxide groups on a silicon wafer can form hydrogen bonds with either -OH or -NH_2_ groups in chitosan (Fig. 3B), which is a natural polysaccharide with abundant -OH and -NH_2_ groups in its structure [144].

Electrostatic self-assembly and LbL assembly techniques rely on electrostatic forces between oppositely charged molecules or surfaces to attach hydrogel coatings. For example, as shown in Fig. 3C, charged functional groups in polymers such as chitosan (-NH_3_^+^) or alginate (-COO^-^, -SO_4_^-^) can interact with oppositely charged substrates, leading to a spontaneous and reversible adhesion [123,132]. LbL assembly (Fig. 3D) builds on the same principle through the sequential adsorption of oppositely charged polyelectrolytes, producing uniform multilayer coatings [133,137,145]. For example, a recent study achieved LbL deposition of polyvinylamine (PVAm) and dopamine-modified hyaluronic acid (HA-DN) via electrostatic interactions on various substrates, including glass, stainless steel, gold, and polyvinyl chloride (PVC) [137]. As shown **in** Fig. 3E and F, successive dip immersion in polymer solutions produced alternating PVAm and HA-DN layers (Fig. 3F), forming a uniform multilayer coating. The LbL assembly technique relies on controlled charge density and deposition sequence to precisely tune coating thickness, surface functionality, and bioactivity. Although electrostatic interactions enable simple multilayer hydrogel construction, these coatings can delaminate under changing environmental conditions. For instance, variations in pH or ionic strength can disrupt electrostatic balance, causing swelling, structural deformation, and interfacial separation in chitosan (CS)/alginate multilayers [146,147]. Similar effects may occur in physiological environments, where local pH shifts during inflammation or tissue remodeling can weaken electrostatic adhesion and reduce coating stability.

Electrodeposition offers an alternative strategy for forming hydrogel coatings by driving charged polymer chains toward an oppositely charged electrode, where deposition occurs through electron transfer and localized pH changes [138,[148], [149], [150]]. Building on this principle, Zhao et al. [138] coated a CS hydrogel containing ibuprofen-loaded mesoporous silica nanoparticles (MSNs) onto a Ti substrate via electrodeposition. As shown in Fig. 3G, the drug-loaded MSNs were dispersed in CS solution, and a Ti cathode with a platinum counter electrode was immersed in the mixture. Applying a negative voltage induced the sol–gel transition of CS and its deposition on the cathode surface, driven by electrochemical water electrolysis and the resulting localized pH gradient [138]. This technique demonstrates the key design principle of electrodeposition—using electrochemical control under mild conditions to achieve precise coating formation and efficient incorporation of bioactive components.

The successful surface attachment of hydrogel coating is usually assessed via surface characterization techniques such as Fourier transform infrared spectroscopy (FTIR) and X-ray photoelectron spectroscopy (XPS) to confirm the presence of specific functional groups associated with the hydrogel coating [136,[151], [152], [153]]. Microscopy techniques and contact angle measurements have also been used to confirm the presence of hydrogel coating on the surface [123,133,154]. However, non-covalent attachment of hydrogel coatings exhibits drawbacks and limitations such as weak coating adhesion and sensitivity to environmental conditions. Non-covalent interactions (e.g., hydrophobic forces, hydrogen bonding) are inherently weaker than covalent bonding, resulting in potential delamination under physiological conditions. In addition, electrostatically attached and physiosorbed hydrogel coatings are susceptible to environmental changes, making them prone to detachment under variations in pH or mechanical stress [133,136]. This instability significantly limits their long-term clinical applications, particularly in medical implants subjected to dynamic physiological environments.

In load-bearing applications such as femoral and dental implants, hydrogel–solid interfaces are typically subjected to substantial mechanical loading (including shear, compressive, and tensile stresses) throughout both implantation and functional use [155]. During surgical insertion, these implants experience large shear and compressive forces that can critically challenge the adhesive integrity between the hydrogel coating and the underlying substrate [155,156]. When the adhesion strength is insufficient, particularly in systems relying on non-covalent interactions—premature delamination of the hydrogel coating may occur even during implantation. Furthermore, dental and orthopedic implants are exposed to continuous cyclic micromovements caused by mastication and locomotion, respectively [157]. These repetitive dynamic forces can progressively degrade the interface, compromising hydrogel adhesion over time and ultimately leading to coating failure and loss of function.

Covalent attachment strategies, which will be discussed in section 3.1.2, offer a promising route to strengthen the bond between hydrogel coatings and implant surfaces. By forming stable covalent anchors, these methods help reduce the risk of coating failure when the implant is exposed to mechanical stress. Regardless of the bonding approach, it is essential to include mechanical testing (such as scratch tests, lap shear assessments, and fatigue loading simulations) as part of the evaluation process [130,158,159]. These tests help ensure that the hydrogel coatings can perform reliably under the types of forces they will encounter in the body [130,159]. Taking these factors into account allows for the development of more robust hydrogel-coated implants that are better suited for the complex mechanical environment of real-world clinical use.

#### 3.1.2 Covalent bonding

Covalent bonding of hydrogels involves permanent chemical linkages between the hydrogel and the surface, resulting in a robust coating with long-term stability, durability, and resistance to delamination under physiological conditions. Strategies developed for the covalent immobilization of hydrogels include Schiff base reactions [160,161], Michael addition [162,163], click chemistry [[164], [165], [166], [167]], silane coupling [[168], [169], [170]], surface-initiated polymerization [152,[171], [172], [173], [174], [175]], and plasma-induced attachment or grafting [174,176,177]. Each of these strategies involves specific mechanisms, which are explained in this subsection.

Covalent attachment of hydrogels can be achieved via reactions between functional groups on the substrate surface and those in the chemical structure of hydrogels. For covalent attachment using a Schiff base reaction (Fig. 4A), primary -NH_2_ groups in the hydrogel structure (e.g., in CS or gelatin) react with an aldehyde (-CHO) or ketone (-C=O)-functionalized surface, resulting in a dynamic imine (-C=N) bond. Then, the formed -C=N groups can be reduced into a stable secondary amine (-C-NH-) bond, which is irreversible, using NaBH_4_ or NaCNBH_3_. For example, a hybrid hydrogel composed of CS and polydopamine (PDA) was attached covalently onto polymethyl methacrylate substrates functionalized with -CHO groups [160]. Schiff base reactions between -NH_2_ groups of either CS or pDA and -CHO functionalities resulted in a robustly adherent hydrogel coating. If more stable covalent linkages are required, carbodiimide chemistry, primarily using 1-ethyl-3-(3-dimethylaminopropyl) carbodiimide (EDC) and N-hydroxysuccinimide (NHS) can be used as a complementary step. In carbodiimide chemistry, EDC/NHS is used to covert carboxyl (-COOH) groups on the hydrogel or substrate into an NH_2_ -reactive NHS ester that subsequently reacts with -NH_2_ groups, forming an irreversible amide (-CONH-) bond [178,179].

Michael addition, as a fast and irreversible reaction, occurs between a nucleophile and an electron-deficient α, β-unsaturated carbonyl compound under physiological conditions [162,163]. In hydrogels, covalent binding occurs when a soft nucleophile such as a thiol (-SH) or amine (-NH_2_) group, present either on the surface or within the hydrogel, attacks the electron-deficient C=C bond. This typically involves acrylate- or maleimide-functionalized surfaces or polymers. Such a reaction results in a strong C-C or C-N bond, permanently linking the hydrogel to the surface as illustrated in Fig. 4B. Click chemistry encompasses three highly efficient and selective reaction types that enable covalent modification of biomaterials. These reactions provide precise control over interfacial bonding while maintaining mild and biocompatible reaction conditions. The first one is azide-alkyne cycloaddition, which forms stable linkages between azide- and alkyne-functionalized components through a cupper (Cu)-catalyzed process [180,181]. A Cu-free variant, strain-promoted azide–alkyne cycloaddition, employs strained cyclooctyne to achieve similar covalent coupling without the cytotoxicity associated with Cu catalysts [182,183].

Among click chemistry mechanisms, thiol–ene click reaction is most widely used for hydrogel coatings. As illustrated in Fig. 4C, -SH groups on either the surface or hydrogel react with alkene (-C=C) groups under UV or radical initiation to form covalent carbon–sulfur (C–S) bonds [[165], [166], [167]]. For instance, SH-functionalized substrates such as silicon, glass, and gold have been coated with ene-functionalized hydrogels using UV-initiated thiol–ene reactions. These include systems based on poly(N-isopropylacrylamide) (PNIPAM), poly(acrylic acid) (PAA), and poly(sulfobetaine methacrylate-acrylic acid-2-hydroxyethyl methacrylate) (P(SBMA-AA-HEMA)) [166,167]. The resulting coatings showed tunable thickness and strong substrate adhesion through covalent C–S bond formation [166,167]. Click chemistry offers versatile, high-yield covalent coupling under mild conditions, allowing robust and biocompatible hydrogel coating formation.

Silane coupling provides an effective strategy for covalently attaching hydrogels to inorganic surfaces such as glass, silicon, and metals [[168], [169], [170],^184^,^185^]. It relies on functional silane molecules that contain hydrolysable groups (e.g., alkoxy or chloro) and reactive ends (e.g., amine, epoxy, or methacrylate). During coupling, hydrolysis generates silanol (Si–OH) groups that condense with surface hydroxyls to form stable siloxane (Si–O–Si) bonds, anchoring the silane to the substrate. The exposed reactive ends then form covalent linkages with functional groups in the hydrogel precursor [170]. As illustrated in Fig. 4D, aminosilane coupling (e.g., using 3-aminopropyltriethoxysilane, APTES) enables ─NH_2_ groups on the surface to react with epoxy, carboxyl, or aldehyde groups in the hydrogel network via carbodiimide or Schiff base reactions [170,186]. Epoxysilane groups can also form covalent bonds with ─NH_2_ or ─SH groups in hydrogel polymers, while isocyanato (─NCO) silanes react with OH or ─NH_2_ functionalities to generate stable urethane or urea linkages [168,187,188]. These reactions enable strong chemical anchoring of hydrogels to silanized surfaces. Silane coupling has been applied to various substrates, including glass, silicon, ceramics, and metals, using silane coupling agents such as 3-(trimethoxysilyl)propyl methacrylate (TMSPMA) to promote robust hydrogel attachment [170]. Studies have demonstrated that hydrogels such as PAAm, Poly(ethylene glycol) diacrylate (PEGDA), and starPEG–heparin systems—exhibit high interfacial toughness and mechanical stability when covalently bonded to pre-silanized or co-modified surfaces [170,184]. Similarly, gelatin methacrylate (GelMA)-based coatings achieved strong adhesion and durability on medical device materials through silane-mediated bonding to PDA– polyethyleneimine (PEI) interlayers [185].

Surface-initiated polymerization (SIP) enables simultaneous polymerization and covalent anchoring of hydrogel chains onto a substrate, providing precise control over coating thickness, crosslinking density, and functionality [152,[171], [172], [173], [174], [175]]. In this approach, surface pre-activation introduces initiation sites that trigger polymer growth directly from the substrate, resulting in robust and uniform hydrogel coatings (Fig. 4E). Depending on the initiator and monomer chemistry, SIP can proceed via atom transfer radical polymerization (ATRP) [189,190], reversible addition-fragmentation chain transfer (RAFT) [191,192], or photopolymerization [176,193]. For instance, UV-initiated SIP has been used to graft N,N-dimethylacrylamide, PNIPAM, and GelMA-based hydrogels onto polymeric, glass, and metallic substrates [171,172,175]. These coatings demonstrated strong adhesion and retained integrity under fluid flow and swelling conditions, attributed to covalent interfacial bonding and, in some cases, catechol-mediated coordination with titanium oxide (TiO_2_) [194,195]. SIP provides a controllable, substrate-adaptive strategy for producing stable hydrogel coatings, where initiation chemistry and monomer selection dictate interfacial strength, film uniformity, and long-term durability.

Electrosynthesis, or electropolymerization, has also been used to covalently graft hydrogel coatings onto various surfaces [152,173]. In this process, the substrate acts as the working electrode in an electrolyte containing monomers and initiators. The applied voltage drives oxidation or reduction reactions, generating surface-bound radicals that initiate polymer growth and covalent bonding. To enhance the cross-linking density and mechanical stability of the forming hydrogel, additional cross-linkers can be incorporated into the electrolyte [173]. In another study, hydrogel coatings from poly (2-hydroxyethyl methacrylate) (PHEMA) and a copolymer of PEGDA and acrylic acid (AA) (PEGDA–AA) were electrosynthesized on titanium substrates [152]. However, the adhesion strength of these electrodeposited hydrogel coatings was not evaluated, leaving their stability under dynamic physiological conditions unverified.

All the wet chemistry methods, such as click chemistry and silane coupling, discussed so far, while effective in fabricating hydrogel coatings on medical implants, have several limitations [196,197]. These include potential toxicity, inadequate adhesion, complex processing steps, and challenges in controlling coating properties. Click chemistry suffers from slow reaction rates, byproducts, and reliance on specific functional groups, while silane coupling is prone to in vivo degradation and may not replicate native tissue properties. Moreover, the use of organic solvents and harsh reagents raises concerns about scalability, reproducibility, and biocompatibility due to residual chemicals. Multiple complex steps that require organic solvents or harsh chemicals make the scalability and reproducibility of these methods quite challenging. In addition, potential biocompatibility concerns due to unreacted reagents or by-products hinder their practical application in biomedical settings.

**Fig. 5:**
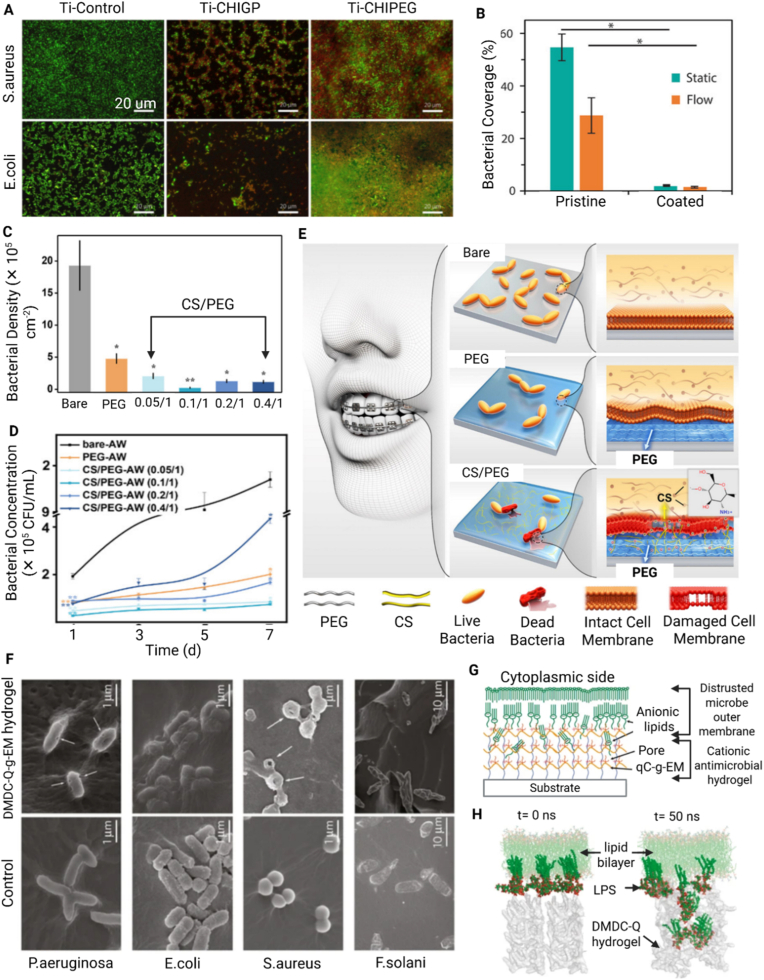
Anti-infection hydrogel coatings. A) Representative fluorescence images (scale bar = 20 μm) of *S. aureus* and *E. coli* bacteria strains on control Ti and hydrogel coated substrates with chitosan hydrogel cross-linked with either genipin (Ti-CHIGP) or polyethylene glycol (Ti-CHIPEG), showing bacteria adhesion and viability. Reporduced with permission from Ref. [199]. Copyright 2023, Elsevier. B) Quantification of *E. coli* (area) coverage of pristine and hydrogel-coated surfaces under static and flow testing conditions. Reproduced with permission from Ref. [171]. Copyright 2020, Wiley. C) Quantification of live *S. mutans* adhesion on bare AW, PEG-coated AW (PEG), and CS/PEG-coated AW surfaces, based on fluorescence microscopy analysis [186], and D) Quantitative evaluations of *S. mutans* bacterial colonies on the same specimens. Reproduced with permission from Ref. [186]. Copyright 2020, ACS E) Schematic illustration of a dental appliance design featuring dual anti-infective functions, showing that PEGylation reduces bacterial adhesion via a surface hydration layer, while the addition of chitosan imparts antibacterial activity to further suppress microbial colonization. Reproduced with permission from Ref. [186]. Copyright 2020 ACS. F) Scanning electron microscopy (SEM) images (scale bar = 100 μm) of various pathogens in contact with DMDC-Q-g-EM hydrogel and control. Reproduced with permission from Ref. [174]. Copyright 2010, Springer Nature. Schematic illustration (G) and computer simulation (H) of the bacteria killing mechanism of DMDC-Q-g-EM hydrogel via an anion sponge’ model, in which the negatively charged bacterial membrane is ‘suctioned’ into the pores of the hydrogel coating and disturbed. Reproduced with permission from Ref. [174]. Copyright 2010, Springer Nature.

To address these challenges, plasma-based methods have emerged, offering a dry, simple, and environmentally friendly alternative for surface modification before the application of hydrogel coatings. Such solvent-free approaches minimize contamination risks and allow for rapid, tunable modifications in a single-step process, making them highly suitable for biomedical applications [174,176,177,198]. Plasma-induced covalent binding of hydrogel coatings involves surface pre-treatment with low-temperature plasma discharge to introduce reactive functional groups (e.g., -OH, -COOH, -NH_2_, or radicals). Such reactive groups are subsequently used to either attach pre-prepared hydrogels or to polymerize directly and graft hydrogel monomers from the plasma-activated surface (Fig. 5F) [174,176,177,198]. For example, thin hydrogel coatings of PEI, poly(N-vinyl pyrrolidone) (PVP), and PAA were immobilized on poly(tetrafluoroethylene) (PTFE) and poly(ethylene terephthalate) (PET) substrates using low-pressure argon plasma treatment [200]. In these systems, covalent attachment occurred through the formation of CONH bonds between the hydrogels and the substrate surface.

In plasma-assisted surface grafting, hydrogel polymeric chains grow and crosslink from monomers pre-immobilized on the surface, resulting in a simultaneous hydrogel formation and covalent bonding onto the surface [174,176,177,198]. Plasma treatment activates the substrate by generating reactive species such as peroxides, which initiate polymerization of acrylate or methacrylate monomers and create strong interfacial linkages. For instance, a hybrid hydrogel composed of quaternized ammonium chitosan and poly(ethylene glycol) methacrylate (PEGMA) was grafted onto a fluoropolymer substrate pre-activated with argon plasma, forming a cross-linked and covalently bonded coating under UV irradiation [174]. Similarly, atmospheric pressure plasma has been used to polymerize and graft acrylate-based hydrogels, such as HEMA and 2-(diethylamino)ethyl methacrylate (DEAEMA), onto gold electrodes via oxygen- and nitrogen-containing functional groups [201].

Plasma immersion ion implantation (PIII) has also been used to create covalently attached hydrogel coatings on polymeric substrates [177]. In PIII, a negative bias voltage is applied to the substrate, resulting in acceleration of positively charged ions in the plasma discharge toward the surface and forming free radicals embedded beneath the surface [202,203]. These radicals migrate to the surface and react with hydrogel precursors or biomolecules, initiating polymerization and forming covalent bonds. [177,204]. For instance, acrylamide- and silk-based hydrogels were grafted onto PTFE substrates pre-treated with PIII to generate surface-embedded radicals [177]. These radicals simultaneously initiated hydrogel polymerization and formed covalent bonds with the substrate, producing strong interfacial adhesion without additional cross-linkers or initiators, as confirmed by T-peel and stability tests.

Overall, non-covalent attachment methods of hydrogel coatings offer simple processing, good biocompatibility, and easy reversibility but are limited by weak interfacial strength and poor long-term stability under physiological loading. In contrast, covalent attachment techniques provide superior mechanical robustness, chemical durability, and resistance to delamination. Although covalent approaches generally require more complex surface functionalization and may face challenges in large-scale uniformity, they offer higher reproducibility and long-term coating stability, making them better suited for demanding biomedical and implant applications.

### 3.2 Hydrogel coating for implants to prevent infections

Infections associated with biomedical implants remain one of the most challenging complications in modern medicine. Despite improvements in surgical procedures and sterilization practices, up to 5% of implants—particularly orthopedic and dental—still become infected [205]. This risk rises even further in high-risk patients or complex surgical cases [206]. These infections are a leading cause of implant failure and place a heavy burden on both patients and healthcare systems. Treatment often involves extended hospital stays, prolonged antibiotic use, and costly revision surgeries. In the U.S. alone, the financial burden of orthopedic implant infections is estimated to be in the billions each year [207]. Beyond economic costs, affected patients endure significant pain, reduced mobility, and emotional distress. This underscoring the urgent need for infection-preventive strategies in implant design and biomaterial development.

Hydrogel coatings have been used to prevent implant-induced infections by forming a protective barrier against bacterial colonization and biofilm formation, either through their intrinsic properties or by releasing antimicrobial agents incorporated into their hydrophilic polymeric networks [148,199]. This section reviews the major classes of anti-infective hydrogel coatings for medical implants, categorized according to their primary material system: (i) CS-based hydrogels, (ii) polyacrylamide (polyAM)-based hydrogels, and (iii) hybrid hydrogel coatings. Each subsection highlights the design principles, functional mechanisms, and biological performance of these hydrogel coatings. A summary of recent studies, including immobilization approaches, stability assessments, and results from both in vitro and in vivo biological evaluations, is provided in Table 1.

**Table 1:** A summary of studies on anti-infection hydrogel coatings, including their immobilization approaches, stability, and in vitro/in vivo evaluations.

Hydrogel coating	Substrate/s	Immobilization approach	Stability evaluations and results	In vitro results	In vivo results	Ref.
Chitosan/alkynyl chitosan	SS wires	Electrodeposition	N/A	The chitosan/alkynyl chitosan hydrogel coating showed higher effectiveness in preventing the growth of *E. coli* and *S. aureus* compared to chitosan hydrogel, as demonstrated by the inhibition zone assays	N/A	[148]
Chitosan	Ti implants	SIP and grafting using either genipin or PEG as crosslinkers	Hydrolytic and enzymatic degradation assays (in the absence and presence of lysozyme) showed that the genipin-crosslinked hydrogel coating exhibits a lower degradation rate than the PEG-crosslinked counterpart	The hydrogel-coated Ti disks showed improved cytocompatibility (with human lung fibroblasts) and reduced haemolytic activity. They also showed significantly reduced *E. coli* biofilm formation, strong contact-killing antibacterial activity against both strains of *E. coli* and *S. aureus*	A 13-week subcutaneous implantation of hydrogel-coated implants showed no adverse effects and minimal tissue response, compared to the control	[199]
Polyacrylamide	PVC and Si tubes	SIP and grafting using DMAA as monomers	Under continuous shear in a rheometer, the hydrogel-coated tubes exhibited a significantly lower coefficient of friction compared to uncoated tubes, indicating enhanced mechanical robustness. Additionally, the hydrogel coating withstood continuous saline flow at physiologically relevant rates (∼1.5 L/min) and pressures (∼100 mmHg) without notable changes in water contact angle or coating thickness	In vitro bacterial adhesion and colonization of *E. coli* were significantly reduced on the hydrogel-coated tubes, incubated in the bacteria media, under both static and flow conditions	The hydrogel-coated tube showed a significant increase in occlusion time, when implanted as an arterial bypass on the iliac artery of Bama pigs	[171]
Polyacrylamide loaded with ZnO NPs	Glass coverslips	Silanization and SIP	N/A	The ZnO NPs-loaded hydrogel coatings demonstrated strong antimicrobial activity against *E. coli*, with bactericidal effects starting at 10 wt% ZnO for thin films (1 μm) and 5 wt% for thick films (4 μm). CFU counts and visual analysis confirmed a ZnO concentration-dependent reduction in bacterial growth, establishing a minimum inhibitory concentration (MIC) of 0.74–1.25 μg/cm^2^	N/A	[172]
Epsilon-poly-l-lysine-graft-methacrylamide	Plastic disks	Plasma treatment and grafting	N/A	The hydrogel-coated disks exhibited antimicrobial activity, evidenced by a logarithmic reduction in viable bacterial and fungal counts	N/A	[176]
Poly (ethylene–glycol diacrylate)-acrylic acid (PEGDA-AA) loaded with CIP	Ti sheets	Electrosynthesis	N/A	The CIP-loaded hydrogel coatings on Ti demonstrated strong antibacterial activity against MRSA, as evidenced by significant inhibition zones in agar diffusion assays, with zone sizes directly correlating with the amount of CIP released, confirming sustained drug delivery and effective antibacterial function	N/A	[152]
PEG and chitosan	SS wire/sheets and Si wafers	Silanization and SIP	The hydrogel coating maintained long-lasting antimicrobial activity over 7 days	In vitro adhesion and colony formation assays using bacterial cultures on the hydrogel-coated substrates demonstrated a 98.8% reduction in bacterial adhesion after 5 hours and a 93.3% suppression of colony formation over 7 days, confirming their short-term antifouling and long-term antimicrobial effectiveness	N/A	[186]

#### 3.2.1 CS-based hydrogel coatings

CS-based hydrogel coatings are among the most widely studied natural materials, recognized for their intrinsic antibacterial and anti-infective properties [199,208]. As a cationic biopolymer derived from the deacetylation of chitin, CS carries a positive charge at physiological pH due to the presence of protonated–NH_2_ groups. This positive charge facilitates electrostatic interactions with the negatively charged microbial cell membranes, leading to membrane disruption and subsequent inhibition of microbial growth [209].

The functional versatility of CS allows it to be used either as a single-component hydrogel [148,199], or in composite formulations with other bioactive agents to tailor antibacterial performance and interfacial properties [210,211]. The fundamental design principle in these coatings is to exploit CS's hydrophilicity and cationic character to both repel bacterial adhesion and induce contact-based bactericidal effects. For example, a composite hydrogel coating composed of CS and alkynyl-functionalized CS has demonstrated effective antibacterial activity against both *Escherichia coli* (*E. coli*) and *Staphylococcus aureus* (*S. aureus*) [148]. The antibacterial performance was evaluated through in vitro assays, including bacterial adhesion tests and zone of inhibition (ZOI) measurements, which confirmed a significant reduction in bacterial colonization. However, the stability or long-term antibacterial performance of the hydrogel coating under conditions that mimic the physiological environment was not assessed [148].

A representative strategy to enhance CS coatings involves crosslinking control, which significantly influences network stability and degradation behavior, contributing to prolonged antibacterial activity and surface integrity under physiological conditions. In one approach, for example, CS hydrogels crosslinked with genipin (CHIGP) formed a more compact polymer network than those crosslinked with PEG (CHIPEG). This denser structure provided greater resistance to both hydrolytic and enzymatic degradation [199]. Both coatings exhibited bacteria-repelling properties and contact killing of *E. coli* and *S. aureus*, as evidenced by live/dead staining assays with SYTO9 and propidium iodide (Fig. 5A). The quantitative fluorescence microscopy analyses showed a higher proportion of dead bacteria on hydrogel-coated surfaces than on uncoated controls. The observed superior antibacterial effect against *E. coli* was attributed to the thinner peptidoglycan layer of Gram-negative bacteria, which is more susceptible to disruption by CS's electrostatic interactions. In vivo subcutaneous implantation of hydrogel-coated and control implants in rats demonstrated that CS-based hydrogel coatings elicited a milder tissue response as verified by histological scores. Combined with their strong in vitro antibacterial performance, these hydrogel coatings exhibit dual functionality, offering effective infection prevention and improved tissue compatibility for biomedical implant applications [199]. However, despite the inclusion of in vivo data, the infection model was not challenged with live bacteria, limiting the conclusions that can be drawn about in vivo anti-infective efficacy of the coatings.

Beyond network design, multifunctionality has emerged as a key trend in CS hydrogel coatings, with recent systems incorporating responsive ion release, antibiotic carriers, or inorganic antibacterial agents to further suppress bacterial colonization and biofilm formation. For instance, CS hydrogels loaded with fosfomycin have shown improved antimicrobial efficacy in preclinical models, highlighting the value of integrating antibacterial payloads within hydrogel matrices to address persistent bacterial populations at implant interfaces [212].

#### 3.2.2 PolyAM-based hydrogel coatings

PolyAM coatings have also been extensively utilized in developing anti-infective surfaces for medical implants, owing to their biocompatibility and tunable physicochemical properties [171,172]. Their high-water content contributes to resistance against protein adsorption and bacterial adhesion, inhibiting bacterial colonization and biofilm formation [213]. In addition to their anti-infection properties, polyAM-based hydrogel coatings exhibit multifunctional biological effects. For instance, an polyAM-based hydrogel was applied to PVC and silicone substrates via surface-initiated polymerization of N,N-dimethylacrylamide (DMA) using Irgacure as a photoinitiator [171]. The resulting ultrathin, highly hydrated coating effectively resisted protein adsorption and bacterial attachment. In vitro assays with green fluorescent protein (GFP)-expressing *E. coli* revealed a pronounced reduction in bacterial adhesion under both static and dynamic flow conditions, even without antibiotic release (Fig. 5B). Furthermore, in vivo evaluation in a porcine iliac artery model showed that hydrogel-coated silicone tubes prolonged occlusion time by 60% compared to uncoated controls, demonstrating the coating's strong antifouling and antithrombogenic performance [171]. This suggests that the developed hydrogel coating not only helps reduce early bacterial colonization but also provides resistance to thrombus formation in blood-contacting environments, reinforcing its potential for use in cardiovascular implant applications. While the study included both in vitro and in vivo experiments, the in vivo model was short-term and did not involve any bacterial challenge during implantation. Therefore, although the coating demonstrated promising antifouling and thromboresistant properties, its actual anti-infective performance under clinically relevant, infection-prone conditions remains to be fully validated. Further long-term implantation and pathogen exposure studies will be necessary to fully characterize the coating's infection-prevention capabilities.

PolyAM-based hydrogel coatings have also demonstrated active antibacterial mechanisms when combined with functional ionic or biopolymeric components. For instance, in a very recent study by Zhang et al., a polyAM/sodium alginate–calcium ions (Ca^2+^) dual-network hydrogel coating was developed for ureteral stents to enhance both anti-infective and mechanical performance [214]. The incorporation of Ca^2+^ ions provided structural reinforcement, while also contributing to bacterial inhibition through ionic modulation. This coating exhibited dual antibacterial mechanisms—a highly hydrated surface that passively prevented bacterial adhesion, and a Ca^2+^–alginate network that actively disrupted bacterial colonization. As a result, it achieved 97–98% inhibition of *E. coli* and *S. aureus*. Moreover, the hydrogel reduced mineral encrustation by 74%, significantly improved lubricity (friction coefficient reduced from 0.19 to 0.06). It also maintained excellent hemocompatibility under in vitro and ex vivo flow conditions simulating the urinary tract. Overall, this work highlights the potential of ionic dual-network polyAM hydrogels to achieve multifunctional anti-infective, anti-fouling, and mechanical advantages in implantable biomedical devices.

Incorporating nanoparticles into polyAM -based hydrogel coatings has emerged as an effective strategy to endow the material with active antibacterial functionality in addition to its inherent antifouling properties. For example, a thermoresponsive PNIPAM hydrogel coating incorporating zinc oxide nanoparticles (ZnO NPs) was fabricated on glass substrates via photopolymerization at various thicknesses [172]. The hydrogel-coated surfaces exhibited sustained release of ZnO NPs, which significantly reduced colony-forming units (CFUs) in bacterial culture assays. This antibacterial activity was primarily attributed to the decomposition of ZnO nanoparticles, resulting in the generation of reactive oxygen species (ROS), which was shown to oxidatively damage the bacterial membranes [215]. The antibacterial performance of the developed hydrogel coating was evaluated entirely through in vitro assays. In these tests, *E. coli* suspensions were incubated on coated glass surfaces for 24 hours, followed by serial dilution and CFU quantification to determine viable bacterial counts. However, the used experimental setup did not account for dynamic flow conditions or include any assessment of compatibility with mammalian cells—both of which are important for evaluating real-world implant performance.

#### 3.2.3 Hybrid hydrogel coatings

Hybrid hydrogels, formed by combining two or more polymers, have emerged as promising coating materials for preventing implant-related infections [152,174,186,216]. In such systems, the incorporated polymers often exhibit synergistic or complementary effects. Typically, one component enhances mechanical strength, stability, or adhesion, while the other contributes biological functionality, such as antifouling, antibacterial, or tissue-interactive properties. These materials can generally be classified into two functional design approaches: (1) hybrid hydrogels integrating multiple polymers without any antimicrobial agents, and (2) hybrid hydrogels embedding antimicrobial polymers or drugs.

Hybrid hydrogel coatings that integrate multiple polymers without any antimicrobial agents rely on synergistic physicochemical effects to achieve antifouling and contact-based antimicrobial performance. For instance, a CS/PEG hydrogel coating exhibited dual functionality, effectively resisting bacterial adhesion while actively suppressing *Streptococcus mutans* (UA159) colony formation [186]. The PEG component formed a hydrated, non-fouling barrier that minimized bacterial attachment, while CS provided electrostatic antimicrobial activity by interacting with the negatively charged bacterial membrane. As shown in Fig. 5C, the hydrogel-coated archwires (AW) exhibited an 88% decrease in bacterial surface coverage. The 7-day CFU assay also confirmed a nearly 90% reduction in viable colonies compared to uncoated samples (Fig. 5D). Among the tested formulations, the optimal CS-to-PEG ratio (0.1:1) achieved the best balance between antimicrobial potency and hydrogel stability. These results support the dual-mechanism model illustrated in Fig. 5E, where PEG contributes antifouling behavior and CS delivers membrane-disruptive bactericidal effects.

In another related study, a dimethyldecylammonium chitosan-graft-poly(ethylene glycol) methacrylate (DMDC-Q-g-EM) hydrogel coating was synthesized on fluoropolymer substrates via surface-initiated polymerization [174]. The developed coating demonstrated strong antimicrobial activity against *P. aeruginosa*, *E. coli*, *S. aureus*, and fungal species such as *F. solani*. Its contact-killing mechanism was attributed to the highly cationic quaternized CS, which acts as an “anion sponge.” It electrostatically extracts anionic membrane components of microbes into the porous hydrogel matrix, leading to membrane rupture and cell death, as illustrated in Fig. 5F–H. In this hybrid structure, the PEG methacrylate segments contributed a hydrophilic and flexible network, which improved surface wettability, mechanical stability, and resistance to nonspecific bio-adhesion. This synergistic combination enhanced both the durability and antimicrobial effectiveness of the coating, underscoring how CS–PEG hybrids can achieve complementary mechanical, antifouling, and biocidal properties.

A second category of hybrid hydrogels enhances antibacterial functionality by embedding antimicrobial monomers or therapeutic agents within a polymeric matrix. One example is the ε-poly-L-lysine-graft-methacrylamide (EPL-MA) hydrogel, which was plasma-grafted onto plastic substrates [176]. This coating exhibited broad-spectrum antimicrobial activity, effectively inhibiting Gram-negative (*E. coli*, *P. aeruginosa*) and Gram-positive (*S. aureus*) bacteria, as well as fungal pathogens (*Candida albicans*, *Fusarium solani*). The antimicrobial mechanism originated from the cationic EPL, which disrupts microbial membranes through electrostatic interactions that compromise membrane integrity [216]. While in vitro CFU and viability assays confirmed strong antimicrobial performance, the tests were limited to static conditions without in vivo validation.

In a separate investigation, a hydrogel composed of PEG diacrylate, and AA (PEGDA-AA) was coated onto Ti substrates via electrosynthesis, with ciprofloxacin (CIP) incorporated either during or after the electrodeposition process [152]. CIP is a broad-spectrum fluoroquinolone antibiotic, commonly used to treat various bacterial infections, including those caused by Gram-negative and some Gram-positive bacteria [217]. The resulting CIP-loaded hydrogel coating effectively inhibited methicillin-resistant *S. aureus* (MRSA), a clinically significant and drug-resistant pathogen [218]. The antibacterial activity was attributed to the hydrogel's ability to retain and release high amounts of CIP, aided by the anionic acrylic acid components. These components enhance drug loading and promote sustained diffusion through ionic interactions with positively charged drug molecules [219]. The antibacterial efficacy was assessed using in vitro methods, including ZOI assays and direct-contact bacterial viability tests, all conducted under static conditions and limited to a single bacterial species.

While numerous studies have reported promising antibacterial effects of hydrogel coatings, a common limitation in the current literature is the predominant use of single-species biofilm models, typically involving well-characterized strains such as *E. coli*, *S. aureus*, or *S. mutans*. Although these models offer reproducibility and control for evaluating antimicrobial performance, they fail to capture the biological complexity of polymicrobial infections commonly encountered in clinical settings. In reality, implant-associated infections are often caused by diverse microbial communities, including anaerobic bacteria, fungi, and antibiotic-resistant strains, which can interact synergistically to enhance biofilm stability, pathogenicity, and resistance to host immune responses [220]. This complexity significantly influences microbial adhesion, biofilm maturation [220], and ultimately the efficacy of antimicrobial coatings. Therefore, while single-species models serve as valuable tools for preliminary evaluation, there is a critical need for more clinically relevant infection models that incorporate polymicrobial biofilms. Without such models, the translational potential and real-world effectiveness of hydrogel-based antibacterial strategies may be overestimated.

## 4 Injectable hydrogels to control infections

Building upon the integration of hydrogels into increasingly sophisticated platforms such as microfluidic implantable devices, injectable hydrogels have also emerged as a promising approach in the development of multifunctional biomaterials. While hydrogel-based microdevices enable precise control over diagnostics and therapeutic release, injectable systems offer distinct advantages in clinical scenarios that demand minimally invasive application, geometric adaptability, and localized therapeutic delivery [221]. These properties are particularly beneficial in the effective management of microbial infections [54]. Such infections include conditions like osteomyelitis, septic arthritis, surgical site infections, and periodontal or peri-implant diseases, which are frequently associated with biofilm-forming pathogens. By adhering to biomaterials or damaged tissues, these pathogens develop protective extracellular matrices that confer resistance to conventional antimicrobial therapies [222]. The chronic nature of these infections frequently results in tissue degradation and irregular defects that require not only antimicrobial intervention but also structural and functional restoration.

In this context, injectable hydrogels have emerged as versatile platforms for localized infection control and tissue regeneration, particularly in anatomically complex or infection-compromised sites [[222], [223], [224]]. Their formulation enables administration in a liquid or semi-solid form, followed by in situ gelation, allowing for conformal filling of irregular defects and prolonged retention at the target site. Through strategic modification of their composition and network architecture, these systems enable the encapsulation of antimicrobial agents and their controlled delivery, achieving high local drug concentrations with minimal systemic toxicity [225,226]. In this context, the performance of injectable hydrogels is strongly influenced by formulation parameters, including polymer composition, concentration, crosslinking density, and the incorporation of responsive or bioactive components.

At the point of care, injectability is governed by viscosity, shear-thinning behavior, and syringeability, which must enable smooth delivery through clinically relevant needle gauges without premature gelation. After administration, gelation kinetics (thermal, photo-, enzymatic, or dynamic-covalent crosslinking) must occur within a clinically practical time window to ensure defect conformability and local retention. Finally, drug-release profiles, driven by diffusion, degradation, affinity interactions, or stimuli-responsiveness, should maintain therapeutic concentrations at the target site while minimizing burst release and systemic exposure. Because infection sites are mechanically and biologically dynamic, these parameters must be jointly optimized with mechanical integrity and adhesion to bridge static in vitro antimicrobial performance into sustained in vivo efficacy. An overview of representative injectable hydrogel systems, their antimicrobial mechanisms, and in vivo performance is provided in Table 2, while detailed descriptions and mechanistic discussions of these studies are presented in the following subsections.

**Table 2:** Representative injectable antimicrobial hydrogel systems and their antimicrobial mechanisms and in vivo outcomes.

System	Hydrogel platform/gelation	Antimicrobial mechanism	Animal model	Key reported in vivo outcome
SNP@PCN@Gel [227]	Injectable hybrid hydrogel; light-responsive activation	PTT/PDT, chemodynamic, and gas/ion therapies	*S. aureus*–infected wound mice model	Reduced bacterial load; accelerated wound closure, epithelial regeneration, collagen formation, neovascularization; no systemic toxicity reported.
CMCS/PEI hydrogel [228]	Physically formed network (hydrogen bonding)	Inherent antimicrobial (cationic PEI-driven membrane destabilization)	Full-thickness wound rat model	Optimized formulation achieved near-complete wound healing by day 14.
CS/β-GP/gelatin thermogel + aspirin + EPO [229]	Thermoresponsive in situ gelation (∼5 min at body temperature)	Dual-phase: early anti-inflammatory (aspirin) + pro-regenerative (EPO)	Ligature-induced periodontitis rat model	Reduced inflammatory markers/osteoclast activity; recovery of alveolar bone height and periodontal structure.
PVA@CS@MTZ (CS@MTZ microcapsules as network component) [230]	Injectable PVA hydrogel crosslinked with CPBA	Controlled antibiotic release (metronidazole); strong underwater adhesion	*P. gingivalis* /Actinomycetes-induced periodontitis rat model	Reduced inflammatory infiltrate and pocket depth, while showing fewer epithelial layers and less hyperkeratosis.
Ag@ZnO heterojunction NPs [231]	Photo-crosslinked injectable hydrogel	Ag^+^/Zn^2+^ release + ROS generation	*S. aureus*-induced diabetic wound rat model	Synergistic antimicrobial action with enhanced healing in infected chronic wound context.
Alginate + CaO_2_ + MnO_2_ nanosheets (PDT-enhancing) [232]	Injectable Ca^2+^-crosslinked alginate network	Relieves hypoxia and enhance ROS generated by PDT	*S. aureus*-induced wound mice model	Reduced inflammation, enhanced collagen deposition, and achieved superior wound closure and skin regeneration.
HA-DA/Fe^3+^/PCN@BP [233]	Photo-initiated, dual-crosslinked injectable hydrogel	PDT/PTT; wet adhesion via catechols	*S. aureus*-induced wound mice model	Reduced bacterial load; improved re-epithelialization and granulation tissue; tunable stiffness and strong wet adhesion.
Chitosan hydrogel + azithromycin [234]	Thermoresponsive chitosan-based gel	Antibiotic delivery against biofilm	Periapical disease rat model	Improved vascularization and tissue regeneration.
CS/Nal-P-113/PDNPs hydrogel [235]	Thermogelling injectable system	Antioxidant, antibacterial and ROS-scavenging activity	Periodontitis-related infection context	Reduced the resorption of alveolar bone after 4 week.
Conductive polyaniline and oxidized dextran hydrogel with amoxicillin [236]	Electroresponsive injectable hydrogel	Voltage- and pH-triggered drug release	Subcutaneous mice model	Showed good biocompatibility and biodegradability.

Abbreviations: BP = black phosphorus; CMCS = carboxymethyl chitosan; CS = chitosan; EPO = erythropoietin; HA-DA = hyaluronic acid–grafted dopamine; MTZ = metronidazole; PCN = porphyrin porous coordination network; PDNPs = polydopamine nanoparticles; PDT = photodynamic therapy; PEI = polyethylenimine; PTT = photothermal therapy; PVA = poly(vinyl alcohol); ROS = reactive oxygen species; SNP = sodium nitroprusside; ZnO = zinc oxide; β-GP = β-sodium glycerophosphate.

### 4.1 Material platforms and synthesis strategies

The performance of injectable hydrogels is critically dependent on the properties of the polymeric matrix and the selected strategy for network formation [237]. These design parameters not only affect injectability and gelation kinetics but also play a pivotal role in determining mechanical strength and the ability to sustain therapeutic delivery. In infected or inflamed environments—often characterized by anatomical complexity—hydrogel properties must be carefully optimized to balance antimicrobial efficacy with tissue compatibility [223,238]. In this context, the selection of appropriate polymers is fundamental to tailoring the physicochemical and biological characteristics of the hydrogel. Natural polymers such as chitosan [238], alginate [239,240], gelatin [241,242], and hyaluronic acid [233,243] are widely employed due to their inherent biocompatibility, injectability, and in some cases, antimicrobial or regenerative bioactivity [244]. Chitosan, in particular, is a biodegradable cationic polysaccharide known for its tunable physical properties and notable inherent antimicrobial activity [245]. It forms shear-thinning solutions under acidic conditions; however, its limited mechanical strength often necessitates reinforcement with additional materials [238,245]. In contrast, synthetic polymers such as PEG and PNIPAM offer precise control over degradation kinetics, stiffness, and chemical functionality [246,247]. PEG-based hydrogels are easily functionalized with antimicrobial agents or peptides and can be crosslinked through light- or chemically-activated methods [248]. PNIPAM is a thermoresponsive polymer that undergoes gelation at body temperature, making it ideal for minimally invasive delivery applications. However, like many synthetic polymers, it exhibits suboptimal biodegradability and lacks intrinsic bioactive cues unless chemically functionalized [225,249].

Semisynthetic hydrogels have been developed to combine bioactivity with mechanical and structural precision to address the limitations of both natural and synthetic systems. A widely studied example is GelMA, which is synthesized by methacrylation of gelatin. This modification enables photocrosslinking under mild conditions while preserving a porous, ECM-like structure that supports cell adhesion and proliferation [242]. However, GelMA injectability and mechanical strength exhibit an inverse relationship related to the polymer concentration, necessitating optimization or reinforcement using nanoparticles or nanogels [241]. Similarly, hyaluronic acid methacrylate (HAMA) retains the favorable biological profile of native hyaluronic acid while enabling tunable degradation and network control via photopolymerization [250]. Though, as hyaluronic acid lacks inherent antimicrobial properties, HAMA-based systems can be functionalized with antimicrobial agents or nanoparticles to enhance their efficacy in the treatment of infections [251]. Overall, functionalization strategies—such as the incorporation of cross-linkable groups [241,251], antimicrobial moieties [252], or responsive elements [253,254]—expand the utility of these hydrogels for infection-responsive, on-demand therapeutic delivery.

Crosslinking methods are also crucial for hydrogel performance, particularly in infection-prone environments [255]. Host–guest systems, particularly cyclodextrins or cucurbiturils, enable reversible, non-toxic gelation and controlled drug release under aqueous conditions [223,225,256]. Despite their advantages, physically crosslinked hydrogels often exhibit limited mechanical robustness and can degrade rapidly under enzymatic or inflammatory stress. To address these challenges, chemical crosslinking strategies are employed to create more durable and stable networks, making them suitable for demanding conditions such as infection. GelMA-LAP systems, in particular, have been applied in dentistry for in situ root canal and periodontal defect filling, supporting antimicrobial delivery and pulp–dentin regeneration [257]. Enzyme-mediated crosslinking, using enzymes like transglutaminase, tyrosinase, or horseradish peroxidase (HRP), provides excellent cytocompatibility and control over gelation time and degradation, depending on enzyme concentration [258]. Altogether, these material choices and synthesis strategies facilitate the design of injectable hydrogels that integrate targeted delivery, stimuli-responsiveness, mechanical robustness, and infection-specific functionality.

Beyond conventional synthetic and natural polymer hydrogels, **decellularized extracellular matrix** (dECM)–based injectable hydrogels have emerged as a biomimetic platform that more closely replicates native tissue microenvironments [259]. Their exceptional biocompatibility and malleability facilitate natural cell infiltration and tissue regeneration, making them highly attractive for regenerative medicine. Thanks to their excellent fluidity, these injectable hydrogels can be administered in a liquid state and subsequently gel in vivo, stabilizing the target site while serving multiple functions, including structural support, tissue repair, and controlled drug release. Enhancing the mechanical properties of dECM-based gelatin injectable hydrogels via crosslinking has been shown to improve mechanical stability, promote angiogenesis, and enable scarless repair when injected around surgical urethral sites in rabbits, offering a promising strategy for treating post-surgical traumatic urethral strictures [260]**.** Moreover, functionalization of injectable dECM hydrogels with cortical neuron–derived exosomes has been reported to enhance tissue repair and motor function recovery following traumatic spinal cord injury [261]. More recently, Agarwal et al. [262] developed a clinically relevant injectable hydrogel derived from decellularized human peripheral nerves, whose mechanical properties closely mimic native CNS tissue. This hydrogel demonstrated potential for minimally invasive CNS applications, including serving as a carrier for cells or drugs and as a scaffold to support axonal growth.

### 4.2 Design considerations for injectable antimicrobial hydrogels

#### 4.2.1 Antimicrobial mechanisms and agent incorporation

The antimicrobial efficacy of injectable hydrogels is primarily determined by their inherent antimicrobial activity and capacity to deliver therapeutic agents locally. These systems can be broadly categorized into (i) hydrogels possessing intrinsic antimicrobial properties and (ii) hydrogels loaded with antimicrobial agents [225]. Both strategies aim to address the limitations of systemic antibiotic therapy—such as inadequate penetration in infected tissues, rapid clearance, and the risk of resistance development—by enabling localized, sustained, and targeted treatment.

Inherent antimicrobial hydrogels are typically based on cationic or amphiphilic polymers capable of disrupting bacterial membranes. For instance, chitosan exhibits intrinsic antibacterial properties due to its high density of protonated amino groups, which interact electrostatically with the negatively charged bacterial cell walls [245]. The antimicrobial efficacy of chitosan can be further enhanced through quaternization—for example, by converting it to hydroxypropyltrimethyl ammonium chloride chitosan (HACC)—which increases its water solubility and strengthens its electrostatic interactions with bacterial membranes [238,245]. Deng et al. [263]developed an all-natural, injectable, self-healing hydrogel by crosslinking HACC with dialdehyde-modified bacterial cellulose (DABC) via dynamic Schiff base reactions. The resulting hydrogel exhibited strong antibacterial activity against both Gram-negative (*E. coli*) and Gram-positive (*S. aureus*) bacteria, along with desirable properties for wound healing including an ECM-mimicking architecture, biocompatibility, adequate compressive strength, and excellent water retention. Importantly, the enhanced in vitro antimicrobial efficacy was attributed to the combined action of quaternary ammonium and amino groups on the chitosan backbone, which disrupt bacterial membranes, alongside Schiff base linkages, which themselves may exert antibacterial effects. These findings were supported by SEM imaging, which showed substantial bacterial deformation and lysis after 24 hours of hydrogel exposure [263].

In another study, Xin et al., [227] introduced SNP@PCN@Gel, an injectable, light-responsive hybrid hydrogel for treating infections and promoting wound healing via synergistic photothermal, photodynamic, chemodynamic, gas, and ion therapies. SEM revealed extensive morphological damage to *E. coli* and *S. aureus*, including disrupted membranes and cell collapse, particularly under light irradiation. In vivo studies in bacteria-infected mouse wound models demonstrated that the hydrogels effectively reduced bacterial load and significantly enhanced wound closure, re-epithelialization, collagen deposition, and angiogenesis, without inducing systemic toxicity. Similarly, a hydrogel system based on carboxymethyl chitosan (CMCS) and PEI demonstrated strong intrinsic antimicrobial activity without the incorporation of antibiotics [228]. Formed through hydrogen bonding interactions, the CMCS/PEI hydrogel exhibited injectability, biocompatibility, and notable antibacterial efficacy, with in vitro assays showing a significant reduction of *S. aureus* and *E. coli* CFUs in a concentration-dependent manner. The antimicrobial mechanism was primarily attributed to the cationic nature of PEI, which destabilizes bacterial membranes, while CMCS contributes solubility and wound-healing support. In vivo, the optimized 5:5 formulation achieved nearly complete wound closure within 14 days, reinforcing its potential for infection control and tissue repair [228].

In parallel, many hydrogels are engineered to serve as localized drug-delivery platforms for antibacterial agents such as antibiotics [226,234], metal ions [264,265], antimicrobial peptides (AMPs), and photoactivatable compounds [233,266]. These hydrogels form in situ and provide a 3D porous matrix that enables the controlled release of therapeutic agents directly at the infection site, thereby offering several clinical advantages. These include enhancing drug concentration at the target, reducing systemic exposure, and minimizing the risk of antimicrobial resistance [225]. Among these, antibiotic-loaded systems remain the most widely investigated and utilized [225]. For instance, in a recent study, a 3D-printed GelMA hydrogel system incorporating PLGA nanoparticles was developed for the dual delivery of rifampicin and vancomycin—two antibiotics commonly used to treat *S. aureus*-associated implant infections [267]. The GelMA-PLGA formulation enabled sustained and localized antibiotic release for up to 21 days, effectively eradicating wild-type *S. aureus* strains as well as rifampicin- and vancomycin-resistant variants. Notably, the dual-drug delivery platform prevented the emergence of resistance observed in single-antibiotic systems, highlighting its potential in preventing biofilm-associated implant infections [267].

**Fig. 6:**
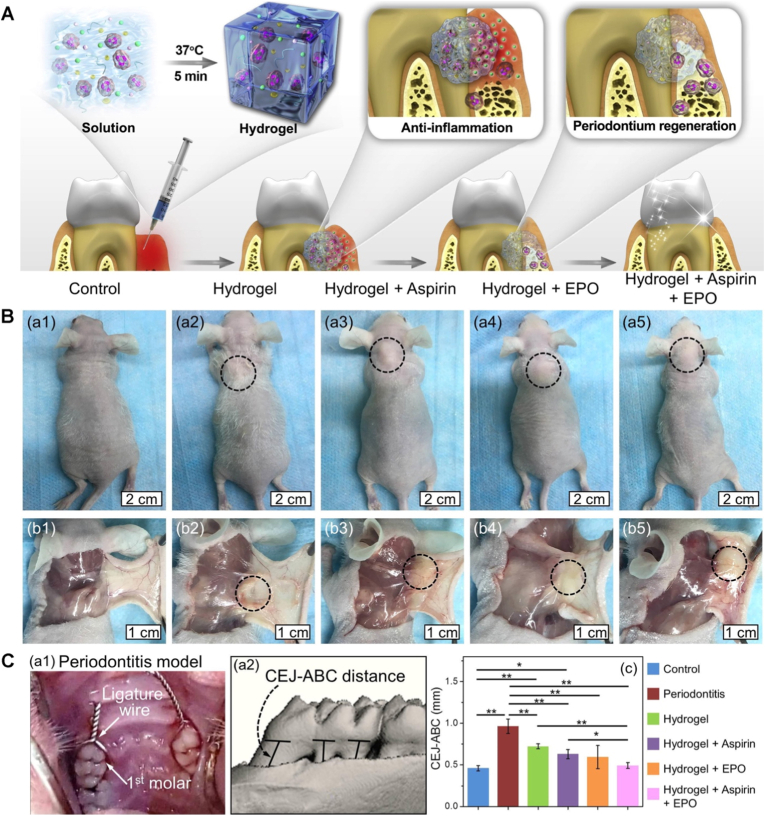
A) Schematic of the thermosensitive CS/β-GP/gelatin hydrogel loaded with aspirin and erythropoietin (EPO), designed for in situ gelation and dual-phase drug release. B) Top - In vivo gel formation and biocompatibility with subcutaneous injection of PBS or pre-gels in nude mice; dotted circles show hydrogel formation after 5 min. Bottom - Skin removed after 2 weeks to reveal remaining hydrogels. C) Micro-CT analysis of alveolar bone regeneration in a ligature-induced periodontitis rat model, with CEJ–ABC distance used to quantify bone loss. Reproduced with permission [229]. Copyright 2019, Elsevier.

Beyond GelMA, other matrix platforms have demonstrated high potential for different infection treatment targets. A notable application of injectable hydrogels in dentistry is the treatment of periodontitis, a chronic inflammatory disease that leads to alveolar bone loss. For instance, to address both infection-induced inflammation and the need for tissue regeneration, a thermosensitive hydrogel composed of chitosan (CS), β-sodium glycerophosphate (β-GP), and gelatin was developed to co-deliver aspirin and erythropoietin (EPO) (Fig. 6A) [229]. This injectable system forms a gel at body temperature within 5 min and supports sustained drug release over 21 days. Aspirin is rapidly released during the early phase to suppress the inflammatory microenvironment by inhibiting COX-2 and MMP-9 expression. In contrast, EPO is gradually released to promote angiogenesis and alveolar bone regeneration, potentially through pathways such as ephrinB2–EphB4 signalling. In vivo studies demonstrated significant reduction in inflammatory markers osteoclast activity, and complete recovery of alveolar bone height and periodontal tissue structure (Fig. 6B and C) [229].

In addition to systems that incorporate antibiotics and anti-inflammatory drugs directly into the hydrogel matrix, increasing attention has been directed toward addressing one of the primary limitations of injectable hydrogels as drug delivery vehicles: their propensity for burst release, particularly with water-soluble agents. While hydrogels are widely recognized as excellent drug carriers due to their high water content, biocompatibility, and tunable porosity, achieving sustained and localized release of certain compounds remains a significant challenge [225]. This is especially true for small, hydrophilic drugs like metronidazole (MTZ), an antibiotic commonly used for anaerobic infections. Due to its moderate water solubility, MTZ tends to diffuse rapidly out of hydrogels, particularly in oral environments where salivary flow can quickly dilute the therapeutic dose. Researchers have explored strategies such as polymer-drug conjugation, drug crystallization, and encapsulation into hydrophobic or physically confining microdomains to slow down diffusion kinetics [225]. Among these, microencapsulation stands out as a versatile and effective approach for controlling the release of MTZ. Recent studies introduced MTZ-loaded chitosan microcapsules (CS@MTZ), which not only enable controlled drug release but also function as a crosslinking component within the hydrogel network [230]. These microcapsules were incorporated into a PVA hydrogel crosslinked with 4-carboxyphenylboronic acid (CPBA), forming an injectable hydrogel (PVA@CS@MTZ) that demonstrates strong underwater adhesion, excellent injectability, and antibacterial efficacy against anaerobic pathogens [230].

Beyond the incorporation of conventional antibiotics, injectable hydrogels have also been developed to deliver metal-based antimicrobial agents [264,265,268], which offer complementary or alternative mechanisms of action—particularly valuable in the context of increasing antibiotic resistance. Silver (Ag^+^) is the most extensively studied metal-based antimicrobial agents [225]. Zhao et al. [264] developed an injectable, self-healing hydrogel (ADCM@Ag) based on carboxymethyl chitosan and aldehyde-functionalized dextran (ADCM), which enabled in situ green synthesis of silver nanoparticles (AgNPs) through Schiff base crosslinking. This system combines strong antibacterial efficacy (>85% bacterial killing) with favorable injectability, shape adaptability, and cytocompatibility. Similarly, a mussel-inspired injectable GelMA/PVA hydrogel incorporating polyzwitterion-stabilized AgNPs demonstrated enhanced antibacterial activity and improved hemocompatibility [265]. Moreover, targeting diabetic wounds, an in situ photo-cross-linkable hydrogel embedded with heterojunction Ag@ZnO nanoparticles achieved synergistic antimicrobial action through dual release of Ag^+^ and Zn^2+^ and reactive oxygen species (ROS) generation [231]. Mechanistically, ROS damage bacteria by inducing oxidative stress, which disrupts cellular components such as membranes, proteins, and DNA. They interact with phospholipids, causing lipid peroxidation, and oxidize proteins, leading to structural and functional impairment. Additionally, ROS can fragment nucleic acids, ultimately triggering cell death through irreversible oxidative damage [231]. Across these platforms, the integration of nanoparticles facilitated controlled and sustained antimicrobial activity while minimizing cytotoxicity, underscoring their potential in the management of infected or chronic wounds.

Building on diverse strategies for delivering antimicrobial agents, including antibiotics, anti-inflammatory drugs, and metal-based nanoparticles. The next frontier in injectable hydrogel design centers on tuning drug release in response to infection-specific cues. Unlike conventional systems driven by passive diffusion or fixed degradation, stimuli-responsive hydrogels enable on-demand release triggered by environmental factors such as pH, redox state, bacterial enzymes, or light [254]. This approach reduces premature drug loss, prolongs therapeutic efficacy, and enhances precision in targeting microbial activity, particularly in biofilm-infected or necrotic tissues. The following section discusses how these systems are engineered to exploit infection-associated microenvironments for controlled release and therapeutic activation.

#### 4.2.2 Stimuli-responsive behaviour for antimicrobial action

The stimuli-responsive behaviour is a cornerstone in the design of injectable antimicrobial hydrogels, enabling on-demand drug release in response to specific biochemical or physical cues associated with infection. These systems may respond to endogenous stimuli, such as acidic pH, elevated ROS, or bacterial enzyme activity, or to exogenous stimuli, such as light, temperature, or magnetic fields [254]. Hydrogels responsive to pH can degrade or swell in acidic microenvironments, which are characteristic of infected and inflamed tissues [254]. Similarly, ROS-responsive hydrogels can trigger the release of antimicrobial agents in environments with elevated oxidative stress, a condition frequently observed in infection sites due to immune cell activity [254].

Among the various stimulus-responsive hydrogels, those responsive to pH and ROS have demonstrated considerable potential for infection-associated bone regeneration. Inflammatory conditions and bacterial activity commonly lead to a localized decrease in pH and an excessive accumulation of ROS, both of which are detrimental to osteoblast function and contribute to delayed bone healing. To address these challenges, Qi et al., [253]developed a smart GelMA-based hydrogel using boronate complexes for the dual delivery of proanthocyanidin—an antioxidant with osteoinductive properties—and amikacin, a broad-spectrum antibiotic. The boronate complexes are cleaved in response to acidic pH and elevated ROS, enabling on-demand drug release within the inflammatory microenvironment. The hydrogel's porous structure, mechanical strength, and biodegradability support tissue regeneration, while its injectability allows for minimally invasive and defect-specific application [253].

Other ROS-based strategies focus not on scavenging but also on generating ROS to achieve potent, broad-spectrum antimicrobial activity, most notably through light-triggered photodynamic approaches [232,233,269]. In this context, photoresponsive injectable hydrogels are capable of localized and on-demand activation of ROS under external light stimuli, enabling spatial control over antibacterial action with minimal systemic toxicity [270]. These photodynamic hydrogels embed photosensitizers (e.g., black phosphorus, Yb_2_O_3_, MoS_2_) that generate bactericidal ROS upon light activation, enabling effective infection treatment even in complex or irregular tissue sites [225,270]. An injectable hydrogel incorporating MnO_2_ nanosheets loaded with a photosensitizer and CaO_2_ nanoparticles within a calcium-crosslinked alginate matrix was developed as a nanozyme-reinforced system [232]. CaO_2_ generates H_2_O_2_, which MnO_2_ converts to O_2_, alleviating hypoxia and enhancing ROS production during photodynamic therapy. This light-activated hydrogel achieved over 99% elimination of *S. aureus* and *E. coli* and significantly reduced biofilm biomass under hypoxic conditions [215]. Using a *S. aureus* biofilm-infected wound model, the hydrogel combined with light significantly reduced bacterial load, promoted wound closure, enhanced tissue regeneration and collagen deposition, and lowered IL-6 and TNF-α levels. However, ROS-based PDT requires high ROS levels for efficacy, posing a risk of oxidative damage to healthy tissue.

**Fig. 7:**
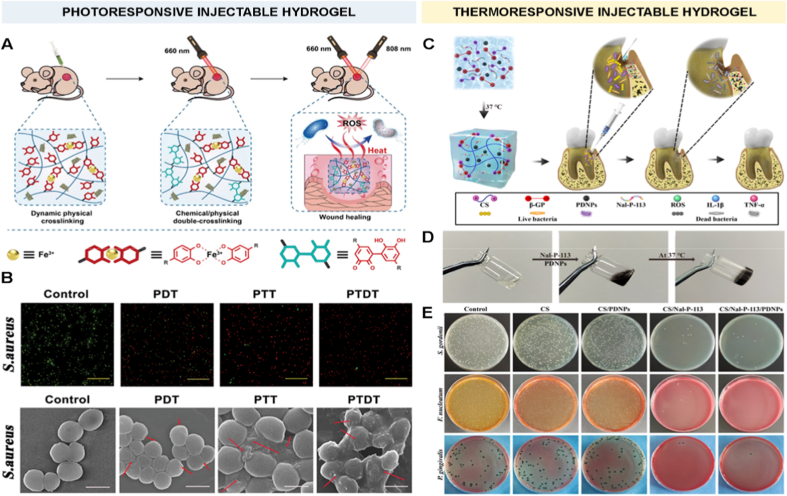
Overview of the antimicrobial performance of photoresponsive and thermoresponsive injectable hydrogels. (A–B) The HA-DA/Fe^3+^/PCN@BP hydrogel features a dynamic physically and chemically double-crosslinked injectable network, offering tunable mechanical properties and synergistic PTT/PDT antibacterial activity that enhances wound healing under laser irradiation (A). Antimicrobial efficacy is evidenced by live/dead staining, which shows increased red fluorescence (dead *S. aureus*) post-treatment, while SEM images reveal extensive bacterial membrane disruption in the PTDT group, with red arrows highlighting ruptured surfaces (B). Reproduced with permission [233]. Copyright 2022, Wiley. (C–E) Similarly, the CS/Nal-P-113/PDNPs hydrogel was engineered to combat oxidative stress, inflammation, and bacterial infection associated with periodontitis (C). Its thermosensitive nature allows it to transition from a liquid at room temperature to a gel at 37 °C, facilitating minimally invasive application (D). The hydrogel's antibacterial effect is further validated by CFU assays, which show substantial reductions in bacterial colony counts following treatment (E). Reproduced with permission [235]. Copyright 2022, Elsevier. (For interpretation of the references to color in this figure legend, the reader is referred to the Web version of this article.)

Combining PDT with mild PTT offers a promising strategy to enhance antimicrobial efficacy while minimizing side effects. Zhang et al. [233] developed a photo-initiated, dual-crosslinked injectable hydrogel (HA-DA/Fe^3+^/PCN@BP) composed of dopamine-functionalized hyaluronic acid and black phosphorus/PCN-224 nanosheets (Fig. 7A). Coordination with Fe^3+^ formed a self-healing, injectable network. Upon 660 nm irradiation, PCN@BP generated ROS that triggered dopamine oxidation, forming covalent pDA crosslinks, reinforcing the hydrogel. Dopamine-derived catechol groups enabled strong adhesion even in moist or irregular tissues. The photo-responsive system allowed tunable stiffness and synergistic ROS/PTT effects, effectively eradicating bacteria. Fluorescence and SEM imaging showed significant membrane damage in *E. coli* and *S. aureus*, especially under combined PDT–PTT. In vitro, the hydrogel showed dose-dependent fibroblast proliferation and enhanced migration (Fig. 7B). In vivo, it significantly reduced bacterial load, promoted reepithelialization, and improved granulation tissue formation in infected wound models, highlighting its therapeutic potential.

Thermosensitive injectable hydrogels feature temperature-triggered sol-gel transition at physiological conditions, allowing in situ gel formation, enabling precise filling of irregular wounds, and prolonging localized retention [254]. For example, a hydrogel composed of Poloxamer 407, lysine, and 3,4-dihydroxyphenylacetic acid (DOPAC) exhibited injectability, rapid gelation at 33 °C, and strong antibacterial and antioxidant properties, achieving 99.77% wound closure by day 14 in a skin defect model. To address the challenge of accessing endodontic root canals, Reis-Prado et al. [234] developed a chitosan-based thermosensitive hydrogel with 3% azithromycin. The hydrogel demonstrated effective action against *E. faecalis* biofilms, cytocompatibility as well as and enhanced vascularization and tissue regeneration in a rat periapical disease model. Similarly, a recently reported chitosan-based hydrogel incorporating antimicrobial peptides (Nal-P-113) and PDA nanoparticles (PDNPs) achieved both antibacterial and antioxidant effects (Fig. 7C) [235]The hydrogel exhibited thermogelling at 37 °C (Fig. 7D), ∼80% ROS-scavenging activity, and ∼99% antibacterial efficacy against *S. gordonii*, *F. nucleatum*, and *P. gingivalis* (Fig. 7E), highlighting its potential for localized periodontitis treatment.

In addition to thermal, pH, and ROS-responsive systems, enzyme- and electrically responsive hydrogels are gaining attention as effective biomaterials for localized therapy. Enzyme-responsive hydrogels are designed to degrade or release their cargo in response to disease-specific enzymes, enabling site-specific activation in infected or inflamed tissues [271]. Electroresponsive hydrogels, on the other hand, incorporate conductive polymers such as polypyrrole or PEDOT, allowing for externally controlled drug release under applied voltage [254]. Importantly, combining multiple stimuli can enhance precision and therapeutic control. For instance, a dual-responsive antibacterial injectable hydrogel was developed based on conductive copolymer polyaniline and oxidized dextran (OD), enabling both voltage- and pH-triggered release of amoxicillin [236]. This system demonstrated an “on-off” pulse release mechanism under electric stimulation and pH-responsive behavior, following a Fickian diffusion model. With tunable gelation time, degradation rate, electroconductivity (up to 0.079 S/m), alongside inherent antibacterial activity and biocompatibility, such dual-responsive hydrogels represent advanced drug delivery systems for tailored treatment of chronic infections [236].

Overall, the design of responsive hydrogels requires a careful balance between sensitivity and structural stability. Premature degradation or uncontrolled release can compromise efficacy and safety, while insufficient responsiveness may fail to activate therapeutic action when needed. Therefore, stimuli-responsive design must be tailored to the infection microenvironment, ensuring that the hydrogel remains inactive until triggered by clinically relevant conditions. When executed effectively, this strategy offers a powerful platform for developing next-generation antimicrobial therapies with high specificity, reduced toxicity, and enhanced regenerative outcomes.

#### 4.2.3 Functional injectability, mechanical and in-situ performance

A key requirement for injectable antimicrobial hydrogels is their functional performance at the target site. This involves easy and site-specific delivery (injectability), rapid and stable gelation after administration, mechanical properties tailored to the local tissue, and strong tissue adhesiveness to ensure retention at the application site. Their injectability is primarily determined by their viscosity, shear-thinning behavior, and gelation kinetics, which must be carefully tuned to ensure smooth extrusion through a syringe while avoiding premature gelation [246]. Shear-thinning properties allow the hydrogel to flow under stress during injection and recover its structure upon reaching the target site, while controlled sol-gel transitions enable in situ solidification, providing mechanical integrity and retention within the defect [223,247]. The rheological profile (e.g., storage modulus, loss modulus, and viscosity over shear rate) is critical for defining usability, with viscoelasticity characterized by a storage modulus (G′) exceeding the loss modulus (G″). These parameters are typically measured through frequency and strain sweep tests to establish the linear viscoelastic region and quantify the hydrogel's stiffness and resistance to deformation [223,247]. Standard protocols typically include assessing viscosity as a function of shear rate to confirm shear-thinning behavior, as well as evaluating gelation kinetics using either the inverted vial test or rheometry under physiological conditions (e.g., 37 °C) [223,246,247,272].

Syringeability and injectability are assessed by measuring the force required to extrude the hydrogel through a syringe—commonly using 18–27 gauge needles—at a constant rate, with acceptable formulations requiring less than 30 N of injection force and ensuring high expulsion efficiency [223,273]. Overall, parameters such as polymer concentration, molecular weight, crosslinking density, and temperature sensitivity play a critical role in tuning gelation properties [223]. For instance, higher polymer concentrations can enhance mechanical strength but may compromise injectability due to increased viscosity, while low concentrations may result in weak or unstable gels [246]. Filimonova et al. [274] found that higher polymer concentrations accelerated gelation and enhanced mechanical properties but also led to greater volumetric shrinkage during processing. Gelation must occur within a clinically relevant timeframe post-injection to avoid burst release, typically triggered by thermal, pH, enzymatic, or chemical stimuli. Delivery conditions also affect hydrogel architecture. For example, Hernandez et al. [272] showed that high extrusion speeds or poor shear-thinning behavior can disrupt microstructure, leading to reduced retention or therapeutic efficacy. Their flow model emphasized the need for accurate high-shear rheological data to ensure predictable performance across varying flow conditions and needle sizes. Mechanical properties such as tensile strength, compressive modulus, and elasticity are critical for ensuring that the hydrogel can withstand physiological stresses without mechanical failure. These parameters are commonly evaluated via compression tests and oscillatory rheology to assess the hydrogel's ability to maintain structural integrity, resist deformation, and match the mechanical environment of the surrounding tissue. This is especially important in dynamic biological settings, such as wound sites or implant environment, where inadequate mechanical performance can lead to early degradation, poor tissue integration, or treatment failure [54].

Injectable hydrogels often suffer from intrinsic softness and instability, limiting their mechanical performance. Various nanoparticles—such as silica, graphene oxide, carbon nanotubes, nanoclays, and nanocellulose—have been incorporated into polymeric matrices to reinforce the network and enhance bioactivity, drug delivery, or hemostatic functions [275]. For example, incorporating rigid CNCs into hydrazone-crosslinked networks increased storage modulus up to 35-fold, improved gelation kinetics, reduced swelling, and maintained cell viability [276]. Gantar et al. [277] showed that adding bioactive glass nanoparticles to Au-(PEGSH)_4_ hydrogels significantly boosted mechanical strength by restricting polymer chain mobility, while preserving self-healing. Similarly, Shi et al. [278] demonstrated that MgSiO_3_ nanoparticles in bisphosphonate-functionalized hyaluronic acid hydrogels enhanced storage modulus and shear resistance through reversible coordination bonds between Mg^2+^ ions and polymer chains. Rheological studies further emphasize that optimal mechanical stability depends on controlled polymer mixing—both insufficient and excessive mixing can compromise network integrity [279].

Self-healing ability, tissue adhesion, and high porosity are critical features that support the functional performance of injectable hydrogels by promoting mechanical resilience and biological integration [225,279]. Adhesion is essential for infection-prone and wound-healing applications and can be markedly enhanced in hydrogels through strategic material design [225]. As reviewed by Mondal et al. [237], effective approaches under moist or dynamic physiological conditions involve incorporating reactive groups—such as aldehydes [280], amines [281], and catechols [282]—to form covalent and non-covalent bonds with tissue surfaces. In addition, catechol groups, inspired by mussel foot proteins, offer strong wet adhesion, while dynamic reversible crosslinking mechanisms such as Schiff base [243] and hydrazone bonds [283] contribute to both cohesive strength and self-healing capabilities [221]. Thus, fine-tuning the crosslinking density and polymer chain architecture—such as the molecular weight between crosslinks—allows control over porosity, swelling, and mechanical robustness. Tavafoghi et al. [239] developed a hybrid injectable hydrogel designed to enhance toughness through ion-induced reversible crosslinking. Combining GelMA with methacrylate-modified alginate (AlgMA) and incorporating divalent cations with photocrosslinkable bonds produced an injectable bioadhesive hydrogel with over 600% greater toughness than GelMA alone, highlighting its promise as a durable and cost-effective tissue sealant.

From a translational standpoint, the mechanical thresholds of injectable hydrogels must be matched to the mechanical demands of the target tissue and the intended clinical function. For non-load-bearing applications, such as soft tissue infections, periodontal pockets, wound beds, or peri-implant defects under minimal mechanical stress, hydrogels with storage moduli typically ranging from ∼0.1 to 10 kPa are sufficient to ensure conformability, tissue adhesion, and biological integration while avoiding stress shielding [284]. In contrast, load-bearing bone-related or implant-adjacent applications, which are exposed to compressive and shear forces, require mechanically reinforced hydrogels with compressive or elastic moduli in the tens to hundreds of kilopascals, particularly when used as temporary space-filling or drug-delivery matrices [284]. Generally, higher water content in hydrogels generally decreases stiffness and strength due to network dilution, but microscale water heterogeneity can enhance energy dissipation and improve mechanical performance. In biomedical contexts, wide variations in tissue water content (≈20–90%) strongly influence mechanical behavior, highlighting the need to consider both macro- and microscale hydration in hydrogel design [285]. Importantly, injectable hydrogels are not intended to function as permanent load-bearing substitutes for bone or cartilage, but rather as bioactive, mechanically supportive interfaces that stabilize the defect environment and control infection.

The translation of in vitro antibacterial or antifouling performance into in vivo efficacy is strongly influenced by these mechanical considerations. Antibacterial activity demonstrated under static in vitro conditions may not be sustained in vivo if insufficient mechanical stability leads to premature degradation, displacement, or dilution of the hydrogel at the target site [286]. Adequate stiffness, cohesive strength, and tissue adhesion are therefore critical to maintain prolonged local residence, preserve effective antimicrobial concentrations, and resist physiological challenges such as fluid flow, tissue motion, and immune-mediated remodeling [54]. Consequently, mechanical integrity acts as a key enabling parameter that bridges in vitro antimicrobial outcomes with in vivo performance, ensuring that antibacterial or antifouling effects are maintained within the clinically relevant time window required for infection control and tissue regeneration.

#### 4.2.4 Biodegradation, biocompatibility, and host interaction

The success of antimicrobial injectable hydrogels in biomedical applications depends on a delicate balance between biodegradability, biocompatibility, and favorable host interaction. The degradation rate must be finely tuned to align with the specific application, whether rapid for acute infections or slower for long-term regeneration, while ensuring that degradation byproducts are non-toxic and non-inflammatory [221,225]. Additionally, hydrogel degradation plays a vital role in tissue regeneration by progressively clearing space for new tissue to grow and integrate, facilitating natural remodeling and functional recovery [221]. The composition of the hydrogel and its implantation environment (e.g., oral cavity, joints, bone defects) influence the degradation profile, which can be modulated using hydrolytic, enzymatic, thiol-exchange, or photolytic mechanisms. Importantly, natural polymers like hyaluronic acid, chitosan, and alginate degrade more rapidly and can be stabilized by crosslinking to prevent premature breakdown, while synthetic polymers often require structural modification or blending to enable appropriate resorption [221,225]. For example, redox-responsive disulfide-crosslinked PEG hydrogels have been engineered to allow precise modulation of degradation kinetics [287]. In vitro, degradation times ranged from 2 to 32 days, depending on polymer concentration: 3% hydrogels degraded within 2 days, whereas 10–15% formulations remained stable for up to 32 days. This degradation rate directly influenced the release kinetics of encapsulated proteins. Incorporation of recombinant human bone morphogenetic protein-2 (rhBMP-2) enabled sustained release during hydrogel degradation, correlating with enhanced ectopic bone formation in vivo [287].

**Fig. 8:**
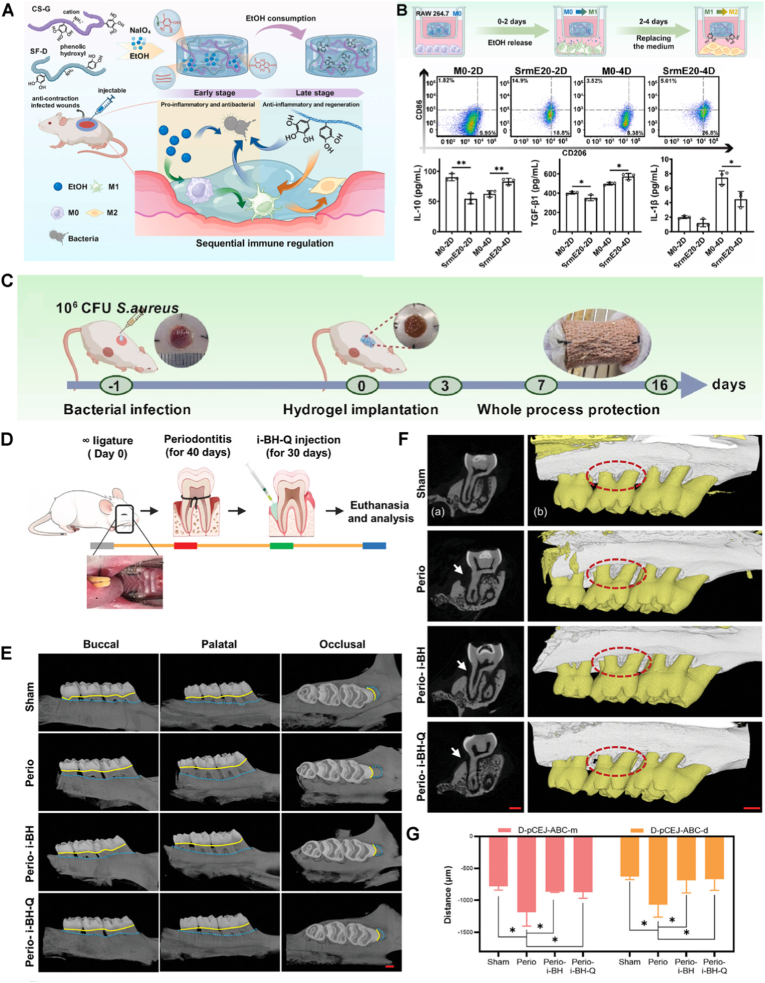
A) Schematic and experimental validation of an injectable hydrogel promoting macrophage polarization (M0→M1→M2) for infection control and tissue regeneration. B) In vitro assays with RAW 264.7 cells assessed phenotypic shifts and cytokine expression. C) Diagram showing the proposed mechanism of wound healing in infected sites with reduced tissue contraction. Reproduced with permission [289]. Copyright 2022, Elsevier. D) In vivo, hydrogel was tested in a rat periodontitis model, with treatment applied to bone defects and healing evaluated via E-G) tissue analysis and micro-CT, including measurement of CEJ–ABC distance and visual comparison of maxillary molars. Reproduced with permission [290]. Copyright 2022, Elsevier.

Hydrogels are generally considered highly biocompatible, particularly when composed of natural polymers or hybrid formulations combining synthetic polymers with biomolecules [288]. These combinations can enhance drug release control, improve mechanical stability, and mitigate inflammatory responses. For example, Wang et al. [289] engineered an injectable hydrogel, SrmE20, to direct macrophage polarization through a sequential transition from M0 to M1 to M2 phenotypes, thereby promoting antibacterial activity and tissue regeneration (Fig. 8A). Initially, ethanol in the formulation promoted M1 polarization and proinflammatory cytokine release (e.g., TNF-α), aiding bacterial clearance. By day 4, its dissipation allowed the hydrogel's cationic and phenolic components to promote M2 polarization, increasing IL-10 and TGF-β1 while reducing IL-1β (Fig. 8B). In infected wounds, this immune modulation accelerated healing via early M2 accumulation, enhanced angiogenesis, and collagen deposition. Immunogenic and antimicrobial hydrogels offer notable therapeutic advantages, particularly against infections, but their design must balance antimicrobial efficacy with biocompatibility [291]. High concentrations of antimicrobial agents within hydrogels can produce reactive byproducts, leading to cytotoxicity and heightened inflammation without proper biological integration [225]. Thus, optimizing composition and release profiles of these agents is essential to minimize adverse effects. Beyond microbial reduction, the intrinsic antimicrobial properties of hydrogels help prevent tissue degradation and modulate immune responses, further enhancing their therapeutic potential.

### 4.3 Bridging laboratory findings to in vivo infection models

The development of antimicrobial hydrogels for implant-associated infections requires thorough evaluation in both in vitro and in vivo models to ensure safety and efficacy. In vitro systems provide controlled conditions to assess antimicrobial activity, enabling manipulation of variables such as bacterial species, biofilm maturity, and hydrogel composition. However, inconsistencies in antimicrobial concentrations and exposure times across studies hinder comparability and the establishment of standardized protocols. Moreover, these models often oversimplify infection dynamics by focusing on single-species biofilms, failing to capture the polymicrobial complexity of implant-associated infections [225]. While advanced tools like next-generation sequencing [292] offer deeper insights into biofilm communities, their use in in vitro studies remains limited. Furthermore, in vitro models lack representation of host immune responses, tissue interactions, and mechanical stresses found in vivo.

In vivo studies are essential for evaluating antimicrobial hydrogels, as they capture the complexities of living systems, including immune responses, tissue integration, and systemic effects. Animal models—from rodents to larger mammals—have been used to assess their efficacy in preventing or treating implant-associated infections [225]. However, selecting an appropriate model is critical, as each presents specific advantages and limitations that must align with the intended clinical application and therapeutic goals. For instance, in wound healing studies, rodent models are frequently utilized due to their manageable size, cost-effectiveness, and well-characterized immune systems. In a representative investigation, Wang et al. [289] employed an anti-contraction full-thickness wound model in mice (diameter = 6 mm), infected with *S. aureus*, to assess the regenerative efficacy of the SrmE20 hydrogel (Fig. 8C). The hydrogel significantly reduced the bacterial burden, accelerated wound closure, and facilitated the restoration of normal skin architecture by day 16. Histological analysis revealed markedly fewer bacteria in tissue sections compared to untreated controls. Another relevant model simulated diabetic wound infections by creating full-thickness *S. aureus*–infected dorsal wounds in streptozotocin-induced diabetic rats [293]. The wounds were treated with a composite system comprising functional core layers and a dECM hydrogel, combined with near-infrared (808 nm) irradiation. This dual treatment approach achieved approximately a 6-log reduction in bacterial load, attenuated inflammation, and significantly enhanced vascularization and collagen deposition, as evidenced by histological and immunohistochemical analyses (e.g., CD31 and type I collagen). These examples underscore the importance of tailored in vivo models—such as infected full-thickness or diabetic wounds—for evaluating hydrogel degradation, drug release kinetics, anti-biofilm efficacy, and regenerative potential under physiologically relevant conditions. Nevertheless, notable differences in skin structure between rodents and humans may limit the direct translatability of findings.

Other specific applications must also be considered when choosing the most appropriate animal infection model, especially in the context of periodontal research. Rodents are frequently used due to their anatomical similarities to human periodontium, ease of handling, and the availability of genetic tools [294]. For example, a rat periodontitis bone defect model was used to assess the regenerative potential of an injectable bioglass hydrogel incorporating quercetin (i-BH-Q system) [290]. This model mimicked the clinical progression of periodontitis through ligature-induced plaque accumulation around the maxillary second molar, which led to localized inflammation, oxidative stress, and alveolar bone resorption (Fig. 8D). This model replicated the clinical progression of periodontitis by inducing plaque accumulation around the maxillary second molar via ligature placement, resulting in localized inflammation, oxidative stress, and alveolar bone resorption (Fig. 8D). Following hydrogel injection into the periodontal defects, maxillary specimens were harvested and analyzed using micro-CT (Fig. 8D–F). The quercetin-loaded hydrogel significantly promoted alveolar bone regeneration, as evidenced by reduced distances between the cementoenamel junction and the alveolar bone crest (CEJ–ABC) compared to untreated periodontitis controls (Fig. 8G) [290]. However, disparities in immune responses, microbiota composition, and overall physiology between animal models and humans may hinder direct clinical translation. In addition, ethical concerns, financial costs, and inter-model variability remain significant limitations in preclinical research.

## 5 Hydrogel implants for bone defects

Bone defects caused by trauma, tumor resection, infection, or degenerative diseases, remain a major clinical and socioeconomic challenge worldwide [295]. Although bone tissue possesses an intrinsic regenerative capacity, this is often insufficient for large or irregular defects, commonly referred to as critical-sized bone defects, which surpass the natural self-healing threshold and require surgical intervention [296]. Autologous bone grafting is still considered the gold standard in bone reconstruction due to its histocompatibility and osteogenic potential; however, the associated limitations—including limited donor availability, donor site morbidity, postoperative pain, and risk of infection—have accelerated the search for synthetic and biomimetic alternatives [296,297]. Allografts and xenografts provide alternatives to overcome donor tissue limitations; however, they carry risks such as immunogenic responses and graft failure. In parallel, emerging bone tissue engineering (BTE) strategies aim to develop functional biomaterials that closely mimic the structural and biological characteristics of native bone [296]. These substitutes must meet stringent requirements: they should physically fill the defect, support osteoconduction, promote osteoinduction and osteogenesis, facilitate vascularization, and ultimately restore the structural and functional integrity of bone tissue [297]. In this context, hydrogels have demonstrated effectiveness not only as scaffolds for cell recruitment and osteogenic differentiation but also as drug delivery systems capable of releasing growth factors (e.g., BMP-2, TGF-β, VEGF) and antimicrobial agents in a controlled and localized manner—features particularly valuable in infected or immunocompromised environments [298].

Beyond their composition (natural or synthetic), the therapeutic efficacy of hydrogel-based implants is closely linked to their functional design. Ideal hydrogel scaffolds for bone regeneration must exhibit osteoconductive architectures that support cellular infiltration and matrix deposition, osteoinductive characteristics to stimulate stem cell differentiation into osteoblasts, and angiogenic properties to restore vascular networks that sustain tissue remodeling and mineralization [298,299]. Advances in fabrication technologies such as 3D bioprinting have enabled precise control over hydrogel architecture and spatial patterning, opening new avenues for patient-specific implants and multifunctional scaffold designs [241,300,301]. Furthermore, the advent of 4D printing has introduced stimuli-responsive materials capable of adapting to physiological conditions post-implantation. This innovation offers promising avenues in bone tissue engineering, particularly through the use of shape-memory polymers and hydrogels that facilitate osteogenesis and enhance tissue integration [73,302]. Moreover, 4D printing aligns with the goals of personalized and sustainable medicine, enabling patient-specific, eco-friendly implants that actively participate in the healing process by mimicking the dynamic nature of living tissues [73]. Altogether, the success of hydrogel implants for bone defects depends on the strategic design of materials and architectures, where factors like responsiveness, degradation rate, mechanical strength, and biocompatibility must be precisely tailored to synchronize with the dynamic stages of bone healing and integration [254,299,303].

### 5.1 Designing hydrogel based on biological functionality

To provide a comprehensive understanding of how hydrogels contribute to bone regeneration, this section categorizes current hydrogel platforms based on their primary biological functions: osteoconduction, osteoinduction, angiogenesis, immunomodulation, and responsiveness to external stimuli. Representative materials, functional mechanisms, and in vitro and preclinical outcomes are highlighted.

#### 5.1.1 Hydrogels with osteoconductivity

Osteoconductivity refers to the material's ability to promote new bone growth on its surface, which is influenced by both the biological environment and the interaction with host cells. To enhance this property, BTE hydrogels often incorporate inorganic components like calcium phosphate ceramics and bioactive glass, as well as cell-loaded biomimetic structures [298,303]. These additions contribute to essential mechanical integrity and bioactivity, promoting integration with host bone and supporting regeneration, even under load-bearing conditions [304].

Calcium phosphate-based bioceramics, such as HAp and β-tricalcium phosphate (β-TCP), are widely employed in BTE due to their chemical similarity to bone minerals, excellent osteoconductivity, and biodegradability. However, their clinical utility is constrained by poor mechanical strength and limited osteoinductive potential [299]. Thus, bioceramics have been integrated into hydrogel systems to enhance bone regeneration. For instance, Qiu et al. [305] developed an injectable, photocurable hydrogel based on methacrylated carboxymethyl cellulose (CMC-MA) and spherical HAp, mimicking native bone matrix and conforming to irregular defects. HAp enhanced osteogenesis by providing bioactive adhesion sites that activated integrin-mediated BMP-2/Smad signaling, promoting cell proliferation, osteogenic gene expression, and bone regeneration in a rat cranial defect model. Despite the advantages of HAp incorporation, the composition and behavior of the hydrogel matrix remain critical for optimal performance [305]. In another study, Morais et al. [258] investigated alginate-based hydrogels—alone or combined with chitosan or hyaluronate—as carriers for GR-HAp, demonstrating that hydrogel composition significantly influences physicochemical properties. The Alg/HA hydrogel exhibited the highest degradation rate, favorable swelling, and low extrusion force, supporting its potential for minimally invasive delivery. The study also showed that higher polymer concentrations and stronger ionic crosslinking enhance viscosity and structural integrity, improving bioceramic retention, while lower concentrations and pH-responsive degradation promote swelling and resorption under physiological conditions.

While HAp offers excellent biocompatibility and mechanical support, its low resorption rate can impede complete bone remodeling. Combining HAp with β-TCP in hydrogel-based scaffolds harnesses the complementary benefits of both bioceramics: HAp provides long-term structural support, whereas β-TCP facilitates early osteoconduction and degrades more rapidly in physiological environments, allowing gradual replacement by new bone. The porous structure of GelMA hydrogels further enhances water infiltration and enzymatic degradation, enabling synchronized scaffold resorption and tissue regeneration. Lee et al. [306] demonstrated that incorporating β-TCP into a GelMA-HAp hydrogel improved mechanical strength, supported in vitro stem cell viability and osteogenic differentiation, and promoted bone regeneration in a rat calvarial defect model. This dual-bioceramic strategy shows strong potential for balancing scaffold stability and bioresorption in alveolar bone repair. This was supported by Zhuang et al. [307]who reported that nano-β-TCP/hydrogel scaffolds enhanced BMSC viability and osteogenesis, evidenced by increased ALP activity, mineralization, and upregulation of BMP-2 and p-ERK1/2. The scaffolds established a bioactive microenvironment with elevated intracellular phosphate and ATP levels, essential for initiating osteogenic signaling, with inhibition assays confirming ATP-dependent activation of the ERK1/2 pathway and BMP-2 expression as key mechanisms.

Cell-loaded biomimetic hydrogels represent a pivotal strategy in BTE, integrating scaffolds with stem cells to mimic the structural and functional characteristics of native bone. Among these, GelMA hydrogels are particularly notable due to their excellent biocompatibility and capacity to support cell adhesion and proliferation. However, their intrinsic osteoinductive potential is limited. To overcome this limitation, GelMA hydrogels are often combined with bone marrow-derived stem cells (BMSCs), leveraging the regenerative capacity of stem cells to enhance osteogenesis and improve bone repair outcomes [299]. Li et al. [308] fabricated an injectable GelMA hydrogel loaded with BMSCs, which was crosslinked in situ under UV light after injection into bone defects. The resulting hydrogel demonstrated good cytocompatibility and promoted substantial bone formation, vascularization, and mature tissue organization in vivo.

A key challenge of photoresponsive hydrogel systems lies in their dependence on photoinitiated crosslinking, commonly induced by ultraviolet (UV) light. This method generates free radicals, which can elicit cytotoxic effects, particularly when cells are encapsulated within the hydrogel matrix during UV exposure. Cellular viability may be significantly compromised due to direct photodamage and the accumulation of ROS, especially in dense or thick constructs where light penetration is inadequate and uneven [299,309]. In this context, Goto et al. [309] proposed a safer crosslinking strategy utilizing riboflavin as a photosensitizer activated by visible light. This approach markedly improved cell viability by circumventing the deleterious effects associated with UV irradiation, including DNA damage and oxidative stress. In vitro studies demonstrated that KUSA-A1 cells—a murine mesenchymal stem cell line with strong osteogenic potential—encapsulated in 20% GelMA–riboflavin hydrogels and cultured under osteogenic conditions exhibited enhanced osteocalcin expression and advanced cellular maturation. These findings confirmed the osteoinductive potential of the riboflavin-crosslinked hydrogel scaffold.

#### 5.1.2 Hydrogels with vascularization

Considering that bone is a highly vascularized tissue that relies on a well-organized vascular network for nutrient exchange and metabolic homeostasis, revascularization is a critical factor in treating bone defects. Hydrogels often require the addition of pro-angiogenic and neuroregenerative growth factors to enhance vascularization and support osteogenesis [296,299,310,311]. The incorporation of bioinorganic ions (e.g. Mg^2+^, Cu^2+^, and Si^4+^) into hydrogels has gained attention for promoting both angiogenesis and osteogenesis by acting as essential cofactors or signaling mediators [299]. For instance, Wu et al. [312] developed injectable hydrogels composed of chitosan, silk fibroin, and glycerophosphate, incorporating copper-containing bioactive glass nanoparticles (Cu-BG NPs) designed to release Si, Ca, and Cu ions in a controlled and sustained manner. These ions synergistically promoted osteogenic differentiation of MC3T3-E1 cells and angiogenic activation of endothelial cells by upregulating key markers such as ALP, HIF-1α, and VEGF. The hydrogel's porous structure facilitated cell infiltration, while chitosan modulated Cu ion release, preventing cytotoxicity and maintaining ion levels within a safe and effective range. In a critical-size rat calvarial defect model, an optimized cell-free hydrogel composed of copper-doped bioactive glass (Cu-BG), chitosan (CH), silk fibroin (SF), and glycerophosphate (GP) achieved complete bone defect repair, accompanied by the formation of neovascularized and mineralized bone tissue. Similarly, an injectable hydrogel comprising phosphocreatine-functionalized chitosan and magnesium oxide nanoparticles (CSMP-MgO) demonstrated outstanding regenerative performance [313]. This system enabled sustained Mg^2+^ release, upregulated osteogenic gene expression, and promoted mineralization in MC3T3-E1 preosteoblasts. Furthermore, it significantly enhanced tube formation by human umbilical vein endothelial cells (HUVECs) and facilitated robust new bone formation in a rat critical-size calvarial defect model, underscoring its strong potential for bone tissue engineering applications.

Another concern about bone defects is that they often create a hypoxic microenvironment that impairs osteogenic differentiation and promotes inflammation through increased ROS production. To address this, Niu et al. [314] developed injectable, fast-gelling hydrogels based on NIPAAm copolymers with high oxygen preservation capacity to enhance cell survival and retention in ischemic tissues. The hydrogels solidified rapidly at 37 °C, maintained higher oxygen levels under hypoxic conditions, and supported the survival and proliferation of encapsulated MSCs, unlike control hydrogels with low oxygen preservation. In addition to enhancing cell viability, perfluorocarbon (PFC)-containing hydrogels—specifically PNHAM-PFC5 and PNHAM-PFC10—significantly promoted the paracrine activity of MSCs. This was evidenced by the upregulated expression of angiogenic (PDGFB) and prosurvival (IGF-1) factors over a 14-day period, suggesting a dual role for these hydrogels in supporting MSC survival and enhancing their therapeutic potential under hypoxic conditions [314].

**Fig. 9:**
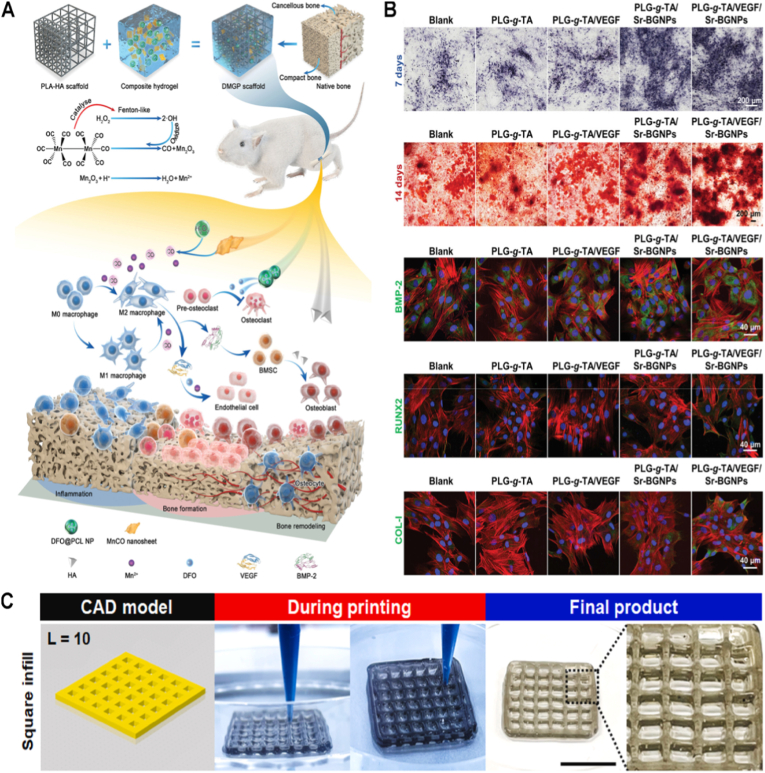
Multifunctional hydrogel-based platforms to treat bone defects. A) Schematic illustration of an osteoimmunity-regulating, biomimetically hierarchical hydrogel scaffold composed of DFO-loaded PCL nanoparticles (DFO@PCL NP), MnCO nanosheets, GelMA (DMGP), and a PLA/HA structural matrix designed to enhance bone regeneration. Reproduced with permission [315]. Copyright 2022, Wiley. B) In vitro osteogenic differentiation induced by the PLG-g-TA/VEGF/Sr-BGNPs hydrogel, demonstrating its bioactivity and potential for bone tissue engineering. Reproduced with permission [310]. Copyright 2024, Wiley. C) 3D printing of complex constructs using GelMA-PPy bioink, highlighting structural integrity and layer-dependent architecture (L indicates the number of printed layers). Reproduced with permission [316]. Copyright, 2023, Elsevier.

Another agent that has been gaining attention is deferoxamine (DFO), an FDA-approved iron-chelating agent, known not only for its pro-angiogenic effect via upregulation of hypoxia-inducible factor-1α (HIF-1α) but also for its immunomodulatory and anti-osteoclastogenic properties; however, its high water solubility can lead to burst release and local toxicity. To address this, Zhang et al. [315] developed a biomimetically hierarchical 3D-printed scaffold composed of deferoxamine-loaded PCL nanoparticles (DFO@PCL NPs), manganese carbonyl (MnCO) nanosheets, GelMA hydrogel, and a polylactide/HAp matrix (DMGP) (Fig. 9A). Within the DMGP scaffold, a Fenton-like reaction between MnCO and endogenous H_2_O_2_ generated Mn^2+^ and CO, further promoting M2 macrophage polarization and synergistically enhancing HIF-1α/VEGF signalling pathways. Gene and protein expression analyses confirmed significantly increased HIF-1α and VEGF levels, resulting in robust neovascularization and efficient healing of critical-size femoral bone defects in vivo. This integrated strategy highlights the potential of combining immunomodulation, pro-angiogenic signalling, and controlled drug delivery in hydrogel design for the repair of large bone defects. Similarly, an injectable and resorbable hybrid hydrogel composed of poly(L-glutamic acid) grafted with tannic acid (PLG-g-TA), VEGF, and strontium-doped bioactive glass nanoparticles (Sr-BGNPs) (PLG-g-TA/VEGF/Sr-BGNPs developed by Huang et al. [310], demonstrated significant angiogenic activity (Fig. 9B). This was evidenced by a tube formation assay using Human Umbilical Vein Endothelial Cells (HUVECs), showing increased numbers of tubes and junctions, as well as greater total tube area and length compared to control groups. These findings confirm that the sustained release of VEGF, in combination with the bioactive ionic components released from Sr-BGNPs, significantly enhances neovascularization—an essential prerequisite for effective bone regeneration.

Importantly, bone repair depends on the sequential and coordinated action of growth factors like VEGF, platelet-derived growth factor-BB (PDGF-BB), and bone morphogenetic protein-2 (BMP-2), which regulate angiogenesis and osteogenesis. To replicate this natural healing cascade, Tang et al. [317] developed a self-healing "sandwich" hydrogel composed of quaternized chitosan and Pluronic® F127, designed to enable controlled, phase-specific release of three bioactive factors. This biomimetic system significantly enhanced vascularized bone regeneration while providing antibacterial protection in a critical-sized (8 mm) calvarial defect model in male Sprague-Dawley rats, underscoring its potential for effective and infection-resistant bone repair. Micro-CT and histological analyses demonstrated that sequential delivery of VEGF, PDGF-BB, and BMP-2 significantly improved bone volume, mineral density, trabecular architecture, and mineral apposition rate resulting in nearly complete defect closure and dense new bone formation at 8 weeks post-implantation.

Building upon the fact that the periosteum plays a crucial role in bone regeneration due to its rich vascularization and osteogenic potential, recent efforts in BTE have focused on developing biomimetic, tissue-engineered periosteum to better replicate the natural healing process. Yu et al. [318] designed a periosteal-bone substitute by incorporating a preosteoblast-derived matrix (pODM) into a GelMA hydrogel, forming a cell-free, engineered membrane that promoted BMSC adhesion, chemotaxis, and osteogenic differentiation. In a rabbit model of critical-sized radial bone defects, the pODM/GelMA scaffold achieved complete bone and medullary cavity regeneration within 12 weeks. Similarly, Yang et al. [319] developed an organic-inorganic biomimetic periosteum (GA/HA-BG) to address the limitations of traditional periosteum-mimicking materials, which often fail to replicate the full biological functionality of native periosteum. Their strategy involved the integration of dopamine-modified gelatin and oxidized hyaluronan with micro/nano bioactive glass (MNBG) to fabricate a co-crosslinked hydrogel membrane that mimics the ECM and exhibits self-adhesive properties to bone tissue. The incorporation of MNBG enhanced membrane stability and enabled the sustained release of bioactive ions (Ca^2+^ and SiO_4_^4−^), thereby promoting osteogenesis and angiogenesis. This was achieved by recruiting stem cells, inducing their osteogenic differentiation, and stimulating VEGF expression in endothelial cells through activation of the PI3K/Akt/HIF-1α signaling pathway.

#### 5.1.3 Hydrogels with osteoinductivity

Bone healing is a complex, multi-stage process regulated by a coordinated network of growth factors that govern cellular proliferation, differentiation, and tissue remodeling. A critical aspect of this process is osteoinductivity—the capacity of a material to recruit progenitor cells and induce their differentiation into osteoblasts [320]. To enhance bone regeneration, the direct application of exogenous growth factors has become a widely adopted strategy. Moreover, the integration of two-dimensional (2D) nanomaterials, such as graphene oxide and black phosphorus, along with bioactive metal ions into scaffold systems, has emerged as a promising approach. These components contribute to the creation of bioactive microenvironments that not only facilitate controlled growth factor release but also enhance osteoinductive potential, promote angiogenesis, and impart antimicrobial properties [299].

Osteoinductive factors play a crucial role in bone healing by activating signaling pathways that regulate bone formation and resorption. Key growth factors like bone morphogenetic proteins (BMPs) and parathyroid hormone (PTH) stimulate osteogenic differentiation—BMPs act via Smad and MAPK pathways to increase Runx-2 and Osx expression, while PTH influences bone mass through endocrine regulation [290]. Incorporating these factors into hydrogels has been reported to enhance their osteoinductive capacity, promoting stem cell differentiation and accelerating bone repair [299]. Diab et al. [321] demonstrated that a BMP-2 delivery system consisting of an electrospun PCL nanofiber mesh tube combined with a silk fibroin hydrogel effectively promoted the repair of critically-sized femoral defects in rats through osteoinduction. The silk hydrogel served as a biodegradable carrier for BMP-2, enabling sustained local delivery, which enhanced osteogenic differentiation and bone formation over 12 weeks, as confirmed by imaging, histology, and improved biomechanical performance. Mechanistically, BMP-2 activated osteogenic pathways by upregulating transcription factors such as Runx-2 and Osx, while the complete degradation of the silk carrier likely facilitated tissue remodeling and bone integration [321]. Furthermore, to restore the osteoblast/osteoclast balance essential for osteoporotic bone healing, Kuang et al. [266] developed an innovative osteoinductive strategy utilizing an injectable hydrogel (DHCP-10PIP/d) engineered for dual-mode, near-infrared (NIR)-triggered delivery of parathyroid hormone (PTH). This system enabled both sustained basal release and on-demand, pulsatile release of PTH upon NIR irradiation, thereby promoting osteogenic differentiation of bone marrow stromal cells (BMSCs) and osteoclastogenesis. The resulting balanced bone remodeling led to significant improvements in bone mineral density, bone volume, and defect healing in an ovariectomized rat model. These findings underscore the potential of spatiotemporally controlled PTH delivery to enhance osteoinduction in compromised bone environments.

Incorporating metal ions into hydrogels has shown promise in enhancing scaffold performance. A 3D-printed composite hydrogel scaffold composed of sodium alginate, gelatin, and α-tricalcium phosphate (α-TCP), supplemented with varying concentrations of strontium ions (Sr^2+^), exhibited notable swelling behavior—expanding up to 600% of its original volume within 4 hours in PBS at 25 °C [240]. Mechanical properties were also improved, with the 0.5 wt% Sr^2+^ group achieving a compressive strength of 2.05 ± 0.13 MPa, compared to 1.78 ± 0.29 MPa in the control. In vitro, BMSCs cultured on these scaffolds demonstrated high viability, enhanced proliferation (CCK-8 assay), and upregulated osteogenic and angiogenic gene expression, particularly in the 0.5 wt % Sr^2+^ group. In vivo, implantation in 5-mm critical-sized rat skull defects showed significantly enhanced bone regeneration after 8 weeks. The 0.5 wt % Sr^2+^ scaffolds achieved the highest bone volume fraction, mineral density, and surface area, accompanied by dense collagen-rich bone formation and increased CD31 expression, indicating both osteogenic and angiogenic enhancement. Nevertheless, although strategies that enable the controlled and sustained release of ions are critical to maintaining their concentration within an optimal therapeutic window and avoiding cytotoxic effects. In this regard, Liu et al. [268] reported a therapeutic approach for periodontitis treatment based on combining the antibacterial and osteogenic potential of zeolitic imidazolate framework-8 (ZIF-8) with the favorable properties of gelatin methacryloyl (GelMA) hydrogel. In this context, ZIF-8, a zinc-based metal-organic framework, enables sustained Zn^2+^ release, promoting antibacterial activity against pathogens like *P. gingivalis* and enhancing osteogenic differentiation through increased alkaline phosphatase activity and mineralization. GelMA, an injectable and photopolymerizable scaffold, mimics the ECM and adapts to irregular periodontal defects. Its arginine-glycine-aspartic acid (RGD) peptide sequence motifs and MMP-sensitive sequences facilitate cell adhesion and remodeling, while UV crosslinking ensures stable in situ localization.

In addition to bioactive hydrogels, there is growing interest in incorporating 2D nanomaterials—such as graphene oxide (GO), black phosphorus (BP) nanosheets, and nanotubes (NTs)—to enhance mechanical and biological performance. Low concentrations of GO improved the structure and mechanical strength of silk fibroin (SF)/GO hydrogels and supported bone marrow stromal cell (BMSC) proliferation and osteogenic differentiation [322]. Similarly, Huang et al. [323] developed a black phosphorus nanosheet (BPN)-based, photo-crosslinked GelMA hydrogel that promotes bone regeneration by releasing phosphate ions, which sequester calcium from the environment to enhance biomineralization and mechanical properties. This system stimulated osteogenesis via the BMP/Runx2 pathway in vitro and significantly improved bone repair in a rabbit model.

While diverse osteogenic materials can elicit potent osteoinductive effects via biochemical signaling and controlled release, yet effective bone regeneration further requires spatial coordination of biological cues with structural and interfacial functions. In complex bone defects, hydrogels must simultaneously support osteogenic differentiation, integrate with native bone, and interface with surrounding soft tissues. This need for spatially resolved functionality has driven the development of Janus hydrogels, which introduce anisotropic architectures capable of delivering distinct biochemical and biomechanical cues within a single construct, an attribute of particular importance in tissue engineering applications [324,325].

Recent Janus-based biomaterials demonstrate how spatial asymmetry can be leveraged to enhance bone regeneration. 3D-printed Janus nanocomposite hydrogels improve mechanical integrity, bioactivity, and stem cell osteogenesis through domain-specific incorporation of osteogenic cues, resulting in superior vascularization and in vivo bone repair compared with isotropic systems [326]. Hierarchically guided Janus hydrogels fabricated by sequential photocuring further integrate a dense protective barrier with a loose osteoconductive phase, enabling space maintenance, controlled degradation, and enhanced osteogenic, angiogenic, and neurogenic responses in bone defect models [327]. Extending this strategy toward dynamic delivery platforms, magnetic Janus micromotors enable targeted stem cell delivery and localized VEGF release, achieving improved angiogenesis and osteogenesis through precise defect-site localization [328].

#### 5.1.4 Stimuli-responsive and 3D-bioprinting hydrogels

3D printing technology has emerged as a potential tool in bone tissue engineering, allowing the fabrication of patient-specific scaffolds with tunable mechanical properties and customizable geometries [240,241,303]. This approach enables precise control over hydrogel volume, shape, and internal architecture, enhancing structural integration and regenerative outcomes [329]. More recently, the integration of stimuli-responsive components into 3D-printed scaffolds has expanded their functionality, enabling on-demand drug delivery, dynamic structural adaptation, and the activation of biological responses at different healing stages [301,302].

Pan et al. [330] developed a microRNA-activated hydrogel scaffold (MAHS) to promote bone regeneration by synchronizing scaffold degradation with the sustained release of osteoinductive microRNA (miR-29b). These scaffolds, fabricated by 3D plotting using gelatin–alginate hydrogels and crosslinked at varying degrees, were designed to gradually degrade while releasing miR-29b complexed with protective gold nanoparticles (miR/NPs), thus mimicking the natural bone healing cascade. The term miRNA-activated scaffold refers to a biomaterial platform that provides structural support and delivers regulatory microRNAs directly to the cellular environment, influencing gene expression to promote osteogenic differentiation. Importantly, a key challenge in designing 3D-bioprinting hydrogels for bone substitutes is addressing the limitations of extrusion-based bioprinting, particularly in structural fidelity and fabrication efficiency. Doyle et al. [331] developed a high-throughput 3D sacrificial printing platform to create reproducible cellular hydrogels containing bioactive particles such as TCP, hyaluronic acid or natural coral. These constructs maintained cell viability, metabolism, and osteogenic differentiation over 7 days, as shown by collagen deposition, calcium uptake, and matrix formation. The platform enabled rapid, non-destructive screening of bone graft materials, offering a scalable, cost- and time-efficient approach for in vitro evaluation.

Considering the critical role of biofunctionality in 3D-bioprinted scaffolds, Zhang et al. [332] developed a multilayered hydrogel scaffold mimicking the zonal architecture of osteochondral (OC) tissue, composed of a cartilage-like top layer (0% nHA), an intermediate calcified cartilage layer (40% nHA), and a subchondral bone layer (70% nHA), all embedded in a robust double-network hydrogel matrix. The gradient scaffold showed improved mechanical properties, with compressive strength up to 900 kPa and enhanced tensile strength. In vivo, BMSC-loaded scaffolds implanted in rat knee OC defects resulted in complete defect filling and formation of hyaline-like cartilage and subchondral bone after 12 weeks. Micro-CT and histological analyses confirmed superior bone regeneration, tissue morphology, and integration. These findings, combined with advances in in situ 3D bioprinting, highlight the translational potential of biofunctional, multilayered constructs for clinical applications. Li et al. [333] demonstrated the potential of in situ 3D bioprinting to repair large segmental bone defects, moving beyond static scaffold fabrication to dynamic, patient-specific treatments performed directly at the injury site. They filled complex defects in swine models with high precision and structural fidelity by combining rapid 3D scanning, reverse-engineering software, and robotic-assisted printing with a bio-ink composed of alginate and PEGDA.

An illustrative example of stimuli-responsive hydrogels is the development of a 3D-printed MXene-integrated composite hydrogel that combines photothermal antibacterial activity and osteogenic capacity to address the complex clinical challenge of infected, irregularly shaped bone defects [301]. Upon NIR irradiation, the MXene component was shown to generate localized heat, effectively eradicating both Gram-positive and Gram-negative bacteria, while the scaffold's composition—including Sr^2+^ ions and β-TCP—supports bone regeneration, demonstrating how external stimuli can be harnessed to trigger therapeutic responses in situ. Beyond light responsiveness, recent advances in 3D bioprinting of conductive hydrogels have enabled the fabrication of increasingly complex biomimetic structures. However, achieving optimal printability and biofunctionality remains a challenge [241,301]. To overcome this, a GelMA-polypyrrole (GelMA-PPy) bioink was developed using a triple cross-linking approach—thermal, photoinitiated, and ionic—which produced a stable, shear-thinning ink with high structural fidelity and cytocompatibility (Fig. 9C) [316]. When combined with electrical stimulation, the bioink significantly enhanced osteogenic differentiation of hBMSCs by activating the NOTCH, MAPK, and SMAD signaling pathways, demonstrating strong potential for bone tissue engineering.

While biomimetic and bioactive scaffolds offer promise for regenerative signaling, recent advances have expanded their application to more complex challenges, such as infection-related bone defects [296,299]. In these cases, promoting osteogenesis alone is insufficient; effective regeneration also requires control of microbial contamination. This has spurred interest in stimuli-responsive scaffolds that provide both osteogenic and antimicrobial effects. A notable example is a 3D-printed hierarchical PEEK scaffold with a pH-sensitive “pDA–Ag–pDA” coating, which releases silver ions in acidic, bacteria-associated environments [334]. This targeted response preserves biocompatibility, eliminates pathogens, and supports bone ingrowth and osseointegration—demonstrating the potential of infection-responsive implants.

### 5.2 Bone defect models for preclinical evaluation of hydrogel implants

**Fig. 10:**
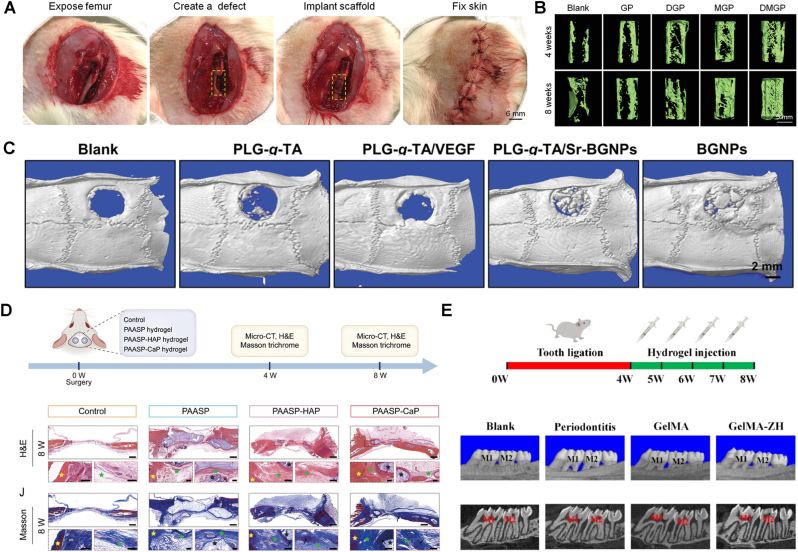
Bone regeneration in critical-sized femoral, calvarial, and periodontal bone defect models. A) Digital images showing scaffold implantation into a 6 mm critical-sized femoral diaphysis defect in Sprague–Dawley rats. B) Representative 3D micro-CT reconstructions demonstrating bone regeneration around femoral defects. Reproduced with permission [315]. Copyright 2022, Wiley. C) 3D reconstructed micro-CT images of calvarial bone following hydrogel implantation in a 5 mm critical-sized cranial defect. Reproduced with permission [310]. 2024, Wiley. D) In vivo bone regeneration in the calvarial defect model. Histological evaluation at 8 weeks post-surgery using H&E and Masson's trichrome staining reveals new bone formation and tissue organization across treatment groups. Reproduced with permission [336]. Copyright 2025, Wiley. E) Ligature-induced periodontal bone defect model. 3D reconstruction images of maxillary alveolar bone at 4 weeks, and corresponding 2D images showing vertical bone loss and regeneration outcomes in different groups. Reproduced with permission [268]. 2022, Elsevier.

Bone defect healing is a complex process governed by cellular, molecular, and mechanical interactions. Animal models play a vital role in assessing bone substitute biomaterials, with a key consideration being whether the defect can heal spontaneously. Critical-sized defects, which do not regenerate naturally within the animal's lifetime, are particularly valuable for distinguishing biomaterial-induced regeneration from natural healing [335]. Common defect sites for bone regeneration studies include the calvaria, femur, tibia, and ulna. Rodents are widely used in early-stage research due to their low cost, ease of handling, and rapid bone turnover. In nude mice, a 3 mm femoral or 4 mm calvarial defect is considered critical-sized [335]. These defects are surgically created under anesthesia with minimal trauma to assess the performance of bone substitute materials. For example, Zhang et al. [315] developed a critical-sized femoral defect model in Sprague–Dawley rats by creating 6 mm × 6 mm cylindrical defects in the femoral diaphysis to evaluate hydrogel-based scaffolds for osteogenesis (Fig. 10A). The scaffolds were implanted into the bone defects, and regeneration was evaluated at 4- and 8-weeks post-surgery using micro-CT, histological analysis, and immunofluorescence (Fig. 10B). Untreated defects exhibited minimal healing and structural deformation. In contrast, defects treated with scaffolds demonstrated significantly enhanced bone formation, increased vascularization, and active tissue remodeling. These outcomes were supported by imaging data and the expression profiles of osteogenic and inflammatory markers.

Another widely used model for evaluating bone regeneration involves the creation of 5 mm full-thickness critical-size defects in the rat calvaria. Huang et al. [310] employed this model to investigate bone regeneration following treatment with various hydrogel formulations. Among the tested groups, the hydrogel incorporating VEGF and strontium-doped bioactive glass nanoparticles exhibited the most effective bone repair, achieving nearly complete defect closure by 8 weeks. Micro-CT analysis revealed significantly increased bone volume and density compared to controls, highlighting the strong regenerative potential of the composite hydrogel (Fig. 10C). Similarly, Zhang et al. [336] utilized the same 5 mm defect model to evaluate the osteogenic efficacy of different hydrogel systems. In their study, 60 μL of hydrogel precursor solution was applied directly into each defect site and photopolymerized in situ using 450 nm blue light for 30 seconds, enabling the hydrogel to conform precisely to the defect geometry and form a stable, integrative scaffold. Using this approach, a calcium phosphate-loaded hydrogel exhibited superior bone regeneration, evidenced by significantly higher surface coverage, bone volume fraction (BV/TV), and bone mineral density (BMD), compared to the control and other hydrogel groups (Fig. 10D). Notably, while the HAp-loaded hydrogel achieved approximately 53% new bone coverage at 8 weeks, the calcium phosphate-loaded hydrogel markedly enhanced bone formation, reaching 88% in a rat cranial defect model.

Despite their widespread use, small animal models possess inherent limitations due to substantial differences in bone remodeling mechanisms compared to humans—most notably, the absence of a fully developed Haversian system. Furthermore, these models typically involve young, systemically healthy animals, which does not accurately reflect the clinical characteristics of patients with critical-size bone defects. In contrast, large animal models—such as rabbits, dogs, goats, and particularly sheep—demonstrate closer physiological and anatomical resemblance to human bone with respect to structure, mechanical loading, and remodeling dynamics. Nonetheless, their use entails significant challenges, including elevated costs, more demanding housing conditions, and heightened ethical considerations.

Dental and craniofacial applications of hydrogel scaffolds necessitate specialized in vivo models to accurately replicate the complex anatomical and pathological conditions of these regions. Periodontal bone defect models, in particular, are commonly established either through ligature-induced periodontitis or surgical procedures to mimic alveolar bone loss around natural dentition. For example, a rat model of periodontal bone loss was developed by placing orthodontic ligation wires around the maxillary first molars, followed by daily subgingival injections of lipopolysaccharide to induce localized inflammation and progressive alveolar bone resorption [268]. After a four-week induction period, the animals were treated with GelMA hydrogels, either alone or loaded with zinc (GelMA-ZH), which were injected directly into the periodontal pockets and polymerized in situ (Fig. 10E). Micro-CT analysis revealed significant alveolar bone resorption and root exposure in the periodontitis group. In contrast, both hydrogel-treated groups showed improved bone preservation, with GelMA-ZH yielding the most pronounced effect. This group exhibited a significantly reduced cementoenamel junction–alveolar bone crest distance (CEJ-ABC) and an increased bone volume/total volume (BV/TV ratio), approaching healthy control levels. These results suggest that GelMA-ZH hydrogel effectively promotes alveolar bone regeneration and mitigates inflammatory bone loss, underscoring its potential for periodontal tissue engineering. Likewise, peri-implant bone defect models are employed to study bone loss around dental implants, effectively simulating peri-implantitis and its treatment strategies [337]. These models facilitate the evaluation of hydrogel-based therapies in infected environments and are particularly valuable for testing multifunctional scaffolds that combine antibacterial, anti-inflammatory, and osteoinductive properties.

Nevertheless, despite their utility in investigating periodontal and peri-implant regeneration, animal models present several limitations. For instance, ligature-induced models, although commonly used, only mimic certain aspects of the disease and fall short of replicating the chronic and multifactorial nature of human conditions [337]. Such models often depend on mechanical plaque retention and, in some cases, inoculation with a single bacterial species—an approach that does not reflect the complex polymicrobial biofilms typical of human periodontitis or peri-implantitis. Consequently, these models may oversimplify host–microbiome interactions and fail to represent the dynamic microbial shifts and ecological diversity observed in clinical settings. While animal models are essential for validating bone substitutes, they pose ethical, financial, and translational challenges. Species-specific differences in bone healing, immune response, and anatomy hinder direct extrapolation to human outcomes. As a result, ex vivo human bone defect models are gaining attention as intermediate platforms. Kluter et al. [338] developed such a model using femoral head specimens from hip replacement surgeries. Cylindrical bone sections (20 mm × 7 mm) with a central defect (6 mm × 5 mm) were filled with collagen type I hydrogel. The model remained viable for 28 days, supporting cell proliferation, migration, and osteogenic marker expression. These systems offer a promising tool for preliminary biomaterial screening, though standardized protocols and further validation are required.

In summary, selecting an appropriate bone defect model should be guided by the target clinical application, the biological question, and the translational relevance of the species and defect site. Small animal models are valuable for initial screening, while large animals and advanced ex vivo systems play a crucial role in bridging toward human application. Integrating these models into the development pipeline of hydrogel-based scaffolds is key to optimizing their performance and accelerating clinical translation.

## 6 Implantable hydrogels for health monitoring

Implantable hydrogel patches are changing the way health is monitored, offering a seamless connection between technology and the human body. These patches, built from soft, water-rich materials, mimic the properties of biological tissues, making them comfortable and effective for long-term use. Scientists have recently enhanced their design, improving conductivity and durability to ensure they provide accurate real-time data. One of the most exciting developments is in cardiac monitoring. Researchers have engineered hydrogel patches that adhere to the heart and transmit electrophysiological signals, aiding in the detection of irregularities and even supporting recovery after myocardial infarction. Similarly, in diabetes management, smart hydrogel patches have emerged as a non-invasive solution for glucose monitoring. Transparent and adhesive, they provide continuous readings without the discomfort of frequent blood testing. These patches are also making strides in neurological applications, where they act as highly sensitive interfaces for detecting brain signals.

### 6.1 Biochemical monitoring

**Fig. 11:**
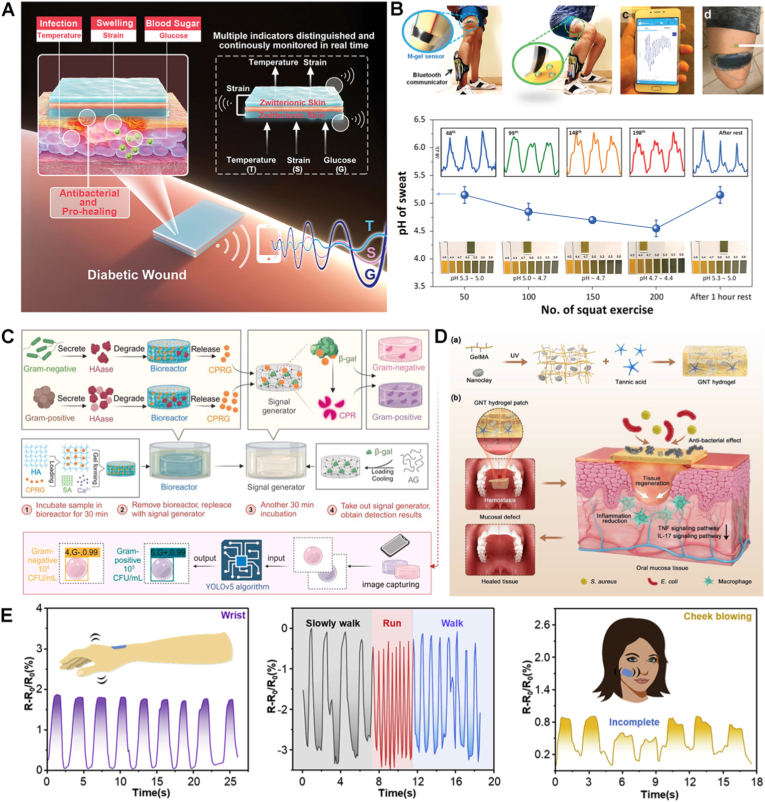
Hydrogels for health monitoring. A) Schematic illustration of a sandwich-structured sensor featuring a multifunctional zwitterionic interface for simultaneous multi-sensing capabilities and accelerated healing of diabetic wounds. Reproduced with permission [339]. Copyright 2021, Wiley. B) Real-time monitoring of sweat during exercise using the M-hydrogel sensor. The sensor was worn on the thigh during squats, with Bluetooth communication via an ankle-mounted device; current changes were displayed on a smartphone; sweat pH was measured through hydrogel resistance changes and validated with pH paper, showing correlation with exercise intensity. Reproduced with permission [340]. Copyright 2021, Wiley. C) Schematic of an AI-assisted smartphone-based colorimetric biosensor using an HA hydrogel bioreactor and agar hydrogel signal generator for bacterial detection, with results analyzed via the YOLOv5 algorithm. Reproduced with permission [341]. Copyright 2024, Elsevier. D) Schematic illustration of GNT hydrogel fabrication and its synergistic hemostatic, antibacterial, and anti-inflammatory effects that promote healing of full-thickness oral mucosal defects [342]. Copyright 2022, American Chemical Society. E) Detection of subtle motions using the hydrogel sensor: resistance signal curves during wrist bending and various locomotion states (slow walking, walking, and running), along with relative resistance changes corresponding to different intensities of cheek blowing [343]. Copyright 2024, Elsevier.

Hydrogel patches have emerged as a promising tool for monitoring a wide range of biological parameters. One of their most impactful applications is continuous glucose tracking in which by incorporating glucose‐sensitive moieties into the hydrogel matrix, these devices deliver real-time, high-precision measurements that simplify daily management for diabetic patients and can significantly improve glycemic control and quality of life. For example, Guo et al. recently reported a zwitterionic thermo–glucose–sensitive “skin-like” sensor featuring a three-layer “sandwich” architecture—two hydrogel sensing layers (zwitterionic carboxylbetaine copolymerized with NIPAAm and MPBA) electrically isolated by an elastomeric film (Fig. 11A). By assigning distinct electrical readouts—|ΔR_0_| for strain, |ΔR_2_–R_0_| for temperature and |ΔR_1_–R_2_| for glucose concentration, their design effectively decoupled cross-talk among signals, enabling continuous, real-time monitoring of infection, swelling and blood glucose at diabetic wound sites [339]. In vivo tests further demonstrated that this conformal patch not only delivered stable multimodal feedback but also accelerated chronic wound closure compared to commercial dressings. While electrical transduction relies on conductivity changes and thermo-responsive mechanics, molecular imprinting offers an alternative sensing strategy by creating template-shaped cavities within the hydrogel for selective analyte capture and optical readout. As such, Giovanninni et al. developed a molecularly imprinted, boronic-acid–functionalized hydrogel that transduced glucose binding into a fluorescence decrease, achieving robust, enzyme-free quantification through imprinting-enhanced specificity rather than electrical sensing [344].

Engineered hydrogel sensors, featuring tailored molecular recognition and multifunctional architectures, also enable sensitive, non-invasive, and multiplexed detection of cancer biomarkers to enhance early diagnosis and improve treatment outcomes [345]. In this sense, a pH‐responsive, tumor‐selective electroconductive hydrogel sensor (CD-PNB@PVA) was engineered so that acidic tumor microenvironments cleave diol–diol crosslinks in carbon-dot nanoparticles, boosting conductivity and producing markedly higher strain and pressure signals in cancer cells versus normal cells [346]. Integrated with a wireless system and smartphone interface, this implantable hydrogel reliably monitored tumor presence in vivo by transmitting real-time sensing data from tumor-bearing mice. This seamless smartphone connectivity not only streamlines data acquisition and user interaction but also exemplifies the shift toward mobile, point-of-care diagnostics. Hu et al. developed a wearable, smartphone-compatible patch embedding an upconversion optical probe within a 3D porous polyacrylamide hydrogel to achieve near-infrared–excited, multiplex chromatic sensing of urea via the inner-filter effect [347].

### 6.2 Physiological and electrophysiological parameters monitoring

Hydrogel sensors for physiological state monitoring harness stimuli-responsive polymers and conductive fillers to transduce changes in temperature, mechanical deformation, and bioelectrical activity into reliable signals, enabling continuous, skin-conformal tracking of key health metrics. Thermosensitive hydrogels, predominantly composed of PNIPAM, undergo a reversible shift from hydrophilic to hydrophobic at their lower critical solution temperature (LCST, ∼34 °C), inducing pronounced volume contraction and optical changes that are ideal for on-demand drug release and precise physiological temperature monitoring [348]. However, during this phase transition PNIPAM networks often suffer from water expulsion, leading to dehydration, adhesion loss, and mechanical weakening, and conventional antidehydration measures, such as polyol or salt additives, only limit surface evaporation without preventing shrinkage. To address these drawbacks, Zhang et al. engineered a gelatin-mesh–reinforced PNIPAM hydrogel (NAGP-Gel), in which the gelatin's helical scaffold traps water molecules and confines polymer-chain movement [348]. The result is the achievement of water retention, volume stability, skin adhesion, and signal reliability across the LCST, overcoming the key limitations of traditional PNIPAM sensors. Muscle‐movement monitoring is another compelling application of hydrogel sensors, as these soft, water‐rich ionic conductors—built from three‐dimensional polymer networks reinforced with conductive nanomaterials—conform seamlessly to skin and transduce mechanical deformation into electrical signals [349]. For example, a recent wearable MXene–polyacrylic acid/polyvinyl alcohol (MXene‐PAA/PVA) hydrogel combined the pH sensitivity of PAA/PVA with the metallic conductivity of MXene nanosheets; bending or stretching the forearm altered the gel's interconnected ion–electron pathways, producing proportional resistance changes, while sweat acidity triggered pH‐driven swelling that reveals muscle fatigue [340] (Fig. 11B). Integrated with a Bluetooth module, this multifunctional patch delivered synchronized electromechanical and biochemical feedback in real time, demonstrating how rational hydrogel design can enable noninvasive, dynamic monitoring of muscle activity.

Building on these conductive-based strategies, hydrogel platforms can likewise serve as soft, conformal electrodes for capturing bioelectrical activity. By embedding conductive networks such as metal nanowires, PEDOT:PSS, or graphene flakes into a hydrated polymer matrix, these hydrogels can record electrophysiological signals like electrocardiogram (ECG) and local field potentials with high fidelity and minimal motion artifact. Among conductive hydrogels, PEDOT:PSS has been reported to combine excellent conductivity, stability, and processability with tunable network structures, which are achieved via crosslinking, ionic or photo‐induced gelation, and versatile fabrication methods (e.g., spinning, printing, molding) to precisely tailor their microstructure and performance [350]. For instance, Yu et al. introduced a PSS‐chain engineering approach in which sodium 4‐styrenesulfonate copolymerized with N-(hydroxymethyl)acrylamide yielded a thermally cross-linkable P(SS-co-NHMAA) matrix that, upon oxidative polymerization with EDOT and subsequent dry-annealing/rehydration, formed highly conductive, soft, tough, and stretchable PSN hydrogels (up to 1850 S m^−1^ conductivity, >50 % strain, ∼4 MPa modulus, ∼400 kJ·m^−3^ toughness). By tuning the PSS/PNHMAA ratio, they precisely modulated electrical, mechanical, and swelling behavior, and, via low-temperature lyophilization/redispersion, produced a 3D-printable bioink for direct-ink-writing of soft skin electrodes, which demonstrated electromyographic recording performance compared to commercial electrodes [351].

### 6.3 Disease detection

Leveraging their tissue‐like softness and tunable chemistry, hydrogel sensors also can enable real‐time disease detection by transducing specific biomarkers and bioelectrical signals, ranging from early infection markers and cardiac electrophysiology to pre‐seizure neural activity, thereby empowering timely diagnosis, personalized treatment strategies, and continuous patient monitoring [352]. To address both infection monitoring and safe antibacterial therapy in wound care, Chen et al. engineered a multifunctional alginate-based hydrogel dressing (Au NCs@AIPH/BTB) that combines real-time pH sensing with synergistic photothermal and free-radical treatment [353]. By embedding bromothymol blue (BTB) within a poly(acrylic anhydride–modified oxidized sodium alginate) network, the dressing visibly reports wound acidity—an indicator of bacterial infection—via color change. Simultaneously, gold nanocages loaded with the stable free-radical generator AIPH serve as both NIR-responsive heat sources and radical producers, in which mild laser irradiation accelerated AIPH decomposition and photothermal heating to deliver potent antibacterial action at lower temperatures, minimizing collateral tissue damage. Both in vitro and in vivo tests confirmed that Au NCs@AIPH/BTB exhibited excellent biocompatibility, effective bacterial eradication, and accelerated wound healing under near-infrared light. Similarly, considering that pathogenic bacteria secrete hyaluronidase (HAase), which degrades hyaluronic‐acid (HA) matrices and serves as a highly specific biomarker for differentiating Gram‐positive from Gram‐negative infections, Cui et al. engineered an AI‐assisted, smartphone‐based colorimetric biosensor comprising a CPRG‐loaded HA hydrogel “bioreactor” and a β-galactosidase–loaded agar “signal generator” [341] (Fig. 11C). Bacterial HAase cleaved the HA matrix in proportion to cell concentration, releasing CPRG that β-gal converts into a visible color change; a custom YOLOv5 algorithm quantified hue shifts from a phone image to infer bacterial load. This simple, instrument‐free assay achieved a 10 CFU/mL detection limit in under 60 minutes, meets WHO's “ASSURED” criteria in food, antimicrobial‐susceptibility, and clinical samples, and exemplifies how combining hydrogel chemistry with AI‐powered imaging can transform point‐of‐care bacterial diagnostics.

Oral‐cavity monitoring demands bioelectronics that adhere in wet, dynamic environments without excessive swelling. Full-thickness oral mucosal defects, for example, is caused by trauma or lesion resection that are plagued by severe bleeding, high infection risk, and slow healing. To address this, Zhu et al. engineered a dual-crosslinked GelMA–nanoclay–tannic acid (GNT) hydrogel, using UV-mediated covalent bonding followed by tannic-acid immersion to create a low-swelling, highly adhesive matrix with rapid hemostasis, potent antibacterial and anti-inflammatory activity [342] (Fig. 11D). In rodent and rabbit models, GNT hydrogels outperformed commercial membranes by accelerating mucosal defect closure, modulating key inflammation pathways, and offering a safe, facile strategy for oral wound repair. In another study, Pan et al. designed a miniaturized, tooth‐adherent RF sensor that used an agarose hydrogel interlayer loaded with AgNPs–chlorhexidine (CHL) between split‐ring resonators to both detect saliva H_2_S—via the formation of Ag_2_S, which shifts the sensor's resonant frequency—and release CHL for on‐site antibacterial therapy [354]. This wireless platform achieved a low detection limit, a broad dynamic range, and high selectivity, accurately distinguishing periodontitis patients from healthy individuals in real saliva samples. In the same context, Hu et al. engineered a structural-color hydrogel by incorporating a disulfide-containing crosslinker (BISS) into a polyacrylamide matrix and templating it as a photonic crystal [355]. They demonstrated real-time, in situ monitoring of VSC production by *P. gingivalis* that correlated with turbidimetric growth curves, and validated exhaled-breath measurements in halitosis patients against clinical diagnoses. By arranging hydrogels of different starting hues into an array, the system distinguished varying oral‐health states for periodontitis risk assessment. Finally, a smartphone app employing colorimetric analysis of captured images enabled rapid, low-cost point-of-care testing of exhaled VSCs, offering a sensitive, user‐friendly screening tool for periodontitis.

### 6.4 Respiratory and sleep monitoring

Hydrogel-based humidity sensors, with their rapid moisture absorption and skin-conformal design, can also enable continuous real-time monitoring of exhaled-air moisture to distinguish breathing patterns and support the management of conditions like sleep apnea, asthma, and pneumonia. For the purpose of accurate monitoring of respiration and body posture, Liu et al. developed a multifunctional wearable sensor using a hydrogel electrolyte composed of PVA, sodium alginate, and starch, enabling dual-mode sensing through resistance and capacitance outputs [343] (Fig. 11E). This design allowed independent detection of mechanical deformation and temperature changes, while also serving as a soft supercapacitor for self-powered operation. The sensor demonstrated excellent sensitivity, durability, and environmental stability, with strain responses up to 2500% and over 90% capacitance retention after 700 cycles. Integrated with a deep learning algorithm, it achieved a 99.259% accuracy in posture recognition and was successfully applied for real-time monitoring of nasal airflow and mechanical vibrations in the neck and hands. Notably, it enabled the non-invasive diagnosis of obstructive sleep apnea syndrome. Although promising, it is important to highlight tat most existing sensors monitor only a single body part or physiological signal, limiting their accuracy and applicability in real-time respiration monitoring.

In this context, wearable electronics have gained significant attention for personal healthcare applications. For example, Liu et al. developed a resistance–capacitance dual-modal hydrogel sensor using a multifunctional cellulose-based hydrogel, offering simultaneous and independent detection of mechanical and thermal stimuli [356]. The sensor was fabricated using a double cross-linked cellulose hydrogel (CH-GT), enhanced with tannic acid, glycerin, water, and NaCl, to improve mechanical toughness and environmental stability. Featuring a dielectric layer sandwiched between two hydrogel layers, the device operates via ionic resistance for temperature detection and parallel-plate capacitance for sensing mechanical vibrations. This allows it to independently monitor nostril airflow, chest and abdomen motion, and arterial pulse. The sensor demonstrated high accuracy and durability in real-time monitoring of obstructive sleep apnea syndrome (OSAS), highlighting its potential as a robust, comfortable, and reliable solution for continuous multimodal respiratory health monitoring.

## 7 Commercial landscape and regulatory considerations

Despite substantial advances in the design of injectable and implantable hydrogel systems, their translation from bench to bedside remains limited, particularly for infection-related and bone-regenerative applications. Although antimicrobial and regenerative hydrogels have demonstrated promising in vitro and preclinical outcomes, relatively few products have achieved regulatory approval or widespread clinical adoption. This discrepancy reflects not only biological complexity but also challenges associated with mechanical reliability, scalability, regulatory classification, and alignment with clinical workflows. One notable exception is the Defensive Antibacterial Coating (DAC®), which has gained clinical traction for localized antibiotic delivery in implant-associated infections [291,357]. However, despite its use in human studies, DAC® is supported by a comparatively limited body of preclinical and in vitro evidence, highlighting a translational gap in which regulatory approval and market implementation have advanced ahead of comprehensive experimental validation [225].

More broadly, injectable hydrogels have been extensively investigated for diverse clinical indications, including facial correction, osteoarthritis, soft tissue regeneration, bone repair, and drug delivery. To date, more than 28 injectable hydrogel-based products have been approved by regulatory agencies such as the FDA and EMA [358]; however, only a small subset is specifically indicated for infection-related applications. This imbalance highlights the additional translational barriers faced by infection-targeting hydrogels, which must simultaneously satisfy antimicrobial efficacy, mechanical stability, biocompatibility, and regulatory safety standards. In this context, DAC®—a hyaluronic acid and poly(lactic acid)–based hydrogel incorporating antibiotics—is currently undergoing clinical evaluation for prosthesis-related infections (NCT04251377) [359]. Most approved hydrogel products remain focused on aesthetic, orthopaedic, or regenerative indications rather than active infection control.

In bone and periodontal regeneration, recent clinical studies highlight the therapeutic feasibility of hydrogel-based systems. RGD-modified hydrogels combined with minimally invasive surgical approaches have demonstrated enhanced bone fill, attachment gain, and increased expression of osteogenic markers such as BMP-2 in periodontal applications [][360]. Similarly, DEXGEL Bone, an injectable composite integrating Bonelike® granules within a dextrin-based hydrogel, has shown safety, ease of use, and efficacy in improving implant stability and promoting bone regeneration during alveolar ridge preservation [361][]. In neurosurgical settings, hyaluronan-based hydrogels, with or without BMP-2 loading, have facilitated cranial bone regeneration in humans with outcomes comparable to autologous grafts and without associated adverse effects^362^ []. Together, these examples highlight the versatility and clinical potential of hydrogel-based platforms across distinct anatomical and therapeutic contexts.

Nevertheless, the overall commercial landscape for hydrogel-based bone substitutes remains restricted. Only a limited number of candidates, such as Gelrin C™, have progressed into clinical trials. In contrast, many widely used hydrogels, including Mebiol® Gel, HyStem®, Corning® Matrigel®, PuraMatrix™, and Biogelx™, are largely confined to preclinical research and in vitro applications due to persistent limitations related to mechanical strength, scalability of manufacturing, and regulatory compliance []. While these materials are valuable for 3D cell culture, drug screening, and biomimetic modeling, insufficient in vivo performance data and the lack of simplified, cost-effective delivery strategies continue to hinder their clinical translation.

Regulatory approval pathways, which vary across regions, generally require extensive and standardized evidence addressing biocompatibility, sterilization, degradation behavior, mechanical performance, and long-term safety [358]. For injectable hydrogel systems intended for bone regeneration or infection control, additional challenges include validating efficacy against clinically relevant biofilms, ensuring reproducible and controllable drug-release kinetics, and confirming the non-toxicity of degradation products. As bioactive and adaptive hydrogel materials continue to evolve, regulatory frameworks are increasingly challenged to accommodate dynamic material–host interactions, immunomodulatory functions, and responsiveness to physiological or pathological cues.

To accelerate clinical translation, future efforts must integrate advances in 3D and 4D printing, bioresponsive crosslinking strategies, and scalable fabrication methods with evolving regulatory standards. Injectable hydrogel systems that combine controlled degradation, reproducible biocompatibility, mechanical reliability, and adaptability to patient-specific conditions, while remaining cost-efficient and compatible with minimally invasive procedures, are more likely to achieve regulatory approval and clinical adoption. Addressing these combined technical, biological, and regulatory challenges is essential to bridge the gap between promising in vitro outcomes and successful clinical implementation of multifunctional hydrogel platforms [300].

## 8 Challenges to clinical translation

Taken together, the diverse hydrogel platforms discussed throughout this review differ substantially in their material composition, crosslinking strategies, functional performance, and readiness for clinical translation. To provide an integrative overview that consolidates these aspects, Table 3 summarizes the hydrogel matrix types discussed in this review—natural, synthetic, and hybrid—highlighting their representative materials, crosslinking methods, key advantages and limitations, typical biomedical applications, and current clinical translation status.

**Table 3:** Comparative overview of natural, synthetic, and hybrid hydrogel matrices, including crosslinking strategies examples, functional performance, typical applications, and clinical translation status discussed in this review.

Hydrogel type	Representative materials	Crosslinking/gelation strategies	Key advantages	Key disadvantages	Typical applications	Clinical translation status	References
**Natural**	Chitosan, alginate, gelatin/collagen, hyaluronic acid, decellularized ECM	Physical, chemical, enzymatic, dynamic covalent, thermal gelation	Biocompatibility; ECM mimicry; inherent bioactivity; good injectability and conformal defect filling	Limited mechanical strength; rapid or uncontrolled degradation; burst release of small hydrophilic drugs; source variability; potential immunogenicity	Injectable infection control; soft tissue regeneration; bone defect fillers in non-load-bearing contexts; wound dressings	Several natural polymers used clinically in regenerative indications; infection- and bone-specific applications remain limited	[[238], [239], [240],^245^,^363^]
**Synthetic**	PEG/PEGDA, PVA, PNIPAM, PAAm, PAA, conductive synthetic backbones (e.g., PEDOT:PSS-based systems)	Free radical polymerization, photo-crosslinking, thermal gelation	Reproducibility; precise control over stiffness, degradation and drug-release kinetics; suitability for stimuli-responsive behavior (pH, ROS, temperature, light, electrical); compatibility with AM and sensing platforms	Limited intrinsic bioactivity; biodegradability concerns for some systems; need for chemical functionalization; potential cytotoxicity from initiators or conductive additives	Stimuli-responsive antimicrobial delivery; 3D/4D printed scaffolds; implantable and wearable hydrogel sensors; drug-delivery matrices	Some synthetic hydrogels approved for non-infectious indications; advanced antimicrobial, bone-regenerative and sensing systems remain preclinical	[105] [225,226,363,364]
**Hybrid**	GelMA, HAMA, PEG–biopolymer blends, alginate–PEG blends, polymer–bioceramic composites, nanoparticle-reinforced systems	Physical, chemical, photo-crosslinking, thermal gelation, dual networks	Combination of bioactivity and mechanical tunability; multifunctionality and suitability for stimuli-responsive behavior; improved retention, adhesion and structural stability; compatibility with injectable, printed and coated systems	Increased formulation and manufacturing complexity; reproducibility challenges; regulatory concerns related to nanomaterials and multifunctional designs	Injectable antimicrobial hydrogels; bone and periodontal regeneration; multifunctional implants; 3D/4D printed scaffolds; advanced coatings and sensing platforms	Predominantly in vitro and preclinical; limited clinical evaluation for bone and infection-related indications	[61,70], [99,253,257,299,363]

Abbreviations: ECM, extracellular matrix; PEG, poly(ethylene glycol); PEGDA, poly(ethylene glycol) diacrylate; PVA, poly(vinyl alcohol); PNIPAM, poly(N-isopropylacrylamide); PAAm, poly(acrylamide); PAA, poly(acrylic acid); PEDOT:PSS, poly(3,4-ethylenedioxythiophene):poly(styrenesulfonate); GelMA, gelatin methacryloyl; HAMA, hyaluronic acid methacrylate; AM, additive manufacturing; ROS, reactive oxygen species.

Overall, the clinical translation of implantable hydrogels (IHGs) is hindered by several critical challenges. One of the primary issues is poor adhesion to medical device surfaces, particularly metallic substrates, due to the mechanical mismatch between the soft hydrogel layer and the rigid implant surface. This mismatch often leads to delamination and detachment of the hydrogel coatings under mechanical stress, especially in high-load-bearing applications such as orthopedic implants and cardiovascular stents. Moreover, many current attachment strategies rely on cytotoxic solvents or chemical agents, which can impede regulatory approval and compromise biocompatibility. Consequently, there is a pressing need for safer and more effective methods to enhance hydrogel adhesion for implantable applications. Plasma technology has emerged as a promising alternative—offering a dry, clean, and environmentally friendly surface modification technique. By tailoring the surface chemistry and topography of the implant material, plasma treatment can significantly improve the adhesion and durability of hydrogel coatings, thereby enabling the development of robust and sustainable solid–hydrogel hybrid systems [117,118,120]. Excessive swelling of hydrogels following coating can compromise their adhesion to implant surfaces and alter their mechanical properties, potentially resulting in delamination or impaired in vivo function. Therefore, precise control of swelling—achieved through modulation of crosslinking density or incorporation of composite materials—is essential to ensure coating stability and maintain overall device performance.

The incorporation of hydrophobic drugs into hydrophilic hydrogel matrices poses a significant formulation challenge due to their limited solubility and poor dispersion. To overcome this, hydrogels are frequently modified to introduce amphiphilic characteristics or incorporate hydrophobic domains capable of encapsulating and releasing poorly water-soluble drugs. For instance, hydrogels grafted with methyl methacrylate or other hydrophobic moieties have demonstrated effective loading and controlled release of such compounds via hydrophobic interactions and domain entrapment [365]. This strategy holds promise for the development of next-generation hydrogel coatings on medical implants, facilitating both robust surface adhesion and localized delivery of hydrophobic therapeutics.

Sterilization also remains a major challenge in the clinical translation of hydrogel-coated medical implants. Conventional sterilization techniques—including autoclaving, gamma irradiation, and ethylene oxide (EtO) exposure—can significantly alter the physicochemical and biological properties of hydrogels [366]. Autoclaving, while effective for microbial inactivation, often causes hydrogel shrinkage, dehydration, and loss of structural integrity due to the high temperature and pressure involved. These conditions drive water out of the polymer network, altering the hydrogel's swelling behavior and mechanical stability, as documented in recent studies on hydrogel sterilization challenges [366]. Gamma irradiation can induce polymer chain scission or excessive crosslinking, leading to changes in swelling capacity, elasticity, and drug-release kinetics [367]. Similarly, EtO treatment, although performed at lower temperatures, may leave residual toxic compounds or trigger chemical modification of functional groups within the hydrogel matrix, affecting biocompatibility and therapeutic performance [366,368].

In addition, different hydrogel and substrate materials can behave differently under sterilization conditions, as their chemical composition, crosslinking density, and interfacial bonding strongly influence thermal and radiation tolerance. In this context, a recent study by Ghosh et al. [369] investigated the stability of nanogel-based hydrogel coatings composed of N-isopropylacrylamide-co-aminopropyl methacrylamide (NIPAM-co-APMA) on various implant substrates, including polypropylene (PP), polyetheretherketone (PEEK), titanium (Ti), and glass. The coatings on polymeric substrates (PP and PEEK) retained their antifouling and antibacterial performance after sterilization with different methods, whereas those on metallic and inorganic surfaces exhibited reduced stability. These discrepancies were linked to differences in surface energy, bonding interactions, and the hydrogel network's sensitivity to sterilization-induced degradation. These findings highlight the necessity for sterilization protocols that are specifically optimized to maintain both the functional integrity and microbiological safety of hydrogel-coated implants.

To address these limitations, emerging low-temperature sterilization methods, such as plasma-based sterilization, ultraviolet (UV) photosterilization, and supercritical CO_2_ (scCO_2_) treatment, are being increasingly explored [370,371]. These techniques offer effective microbial inactivation at sub-thermal conditions, avoiding the heat- and radiation-induced degradation commonly observed in conventional methods.In plasma-based sterilization, reactive oxygen and nitrogen species (ROS and RNS), along with charged particles and UV photons, interact with microbial cell walls, nucleic acids, and membrane lipids, causing oxidative and structural damage that leads to cell death. Importantly, these processes occur at near-ambient temperatures, making them suitable for heat-sensitive hydrogel coatings, while also promoting surface activation that can enhance coating adhesion and hydrophilicity. UV photosterilization achieves microbial inactivation primarily through DNA and RNA damage caused by short-wavelength UV-C irradiation, which induces thymine dimers and disrupts cellular replication. Although UV has limited penetration depth, it provides a rapid, chemical-free disinfection route for thin hydrogel coatings and surface treatments. Meanwhile, supercritical CO_2_ sterilization operates under mild temperature and pressure conditions, combining penetration efficiency with the solvating power of CO_2_ and the optional use of peracetic acid or hydrogen peroxide as co-agents. This method effectively inactivates microorganisms while preserving hydrogel hydration, crosslink density, and mechanical integrity.

Collectively, these low-temperature sterilization approaches achieve high antimicrobial efficacy while minimizing thermal and chemical damage to hydrogel networks. By maintaining both microbiological safety and functional integrity, they hold significant potential to enable the safe clinical translation of hydrogel-coated implants.

## 9 Conclusion and future outlook

Implantable hydrogel coatings (IHGs) have emerged as versatile platforms for biomedical, drug-delivery, and diagnostic applications, supported by advances in material design, bioactive incorporation, and tunable network architectures. Their applicability across multiple tissue targets, including bone, cartilage, skin, and ocular systems, highlights their broad potential. Nevertheless, several key challenges continue to limit their clinical translation. One of the main barriers is the insufficient adhesion of hydrogel coatings to implant surfaces, particularly metallic substrates. The mechanical mismatch between soft hydrogel coatings and rigid implants frequently leads to delamination under physiological loading, especially in high-load-bearing applications. In addition, commonly used attachment strategies often rely on cytotoxic solvents or chemical agents, which may compromise biocompatibility and regulatory acceptance. Plasma-based surface modification has emerged as a promising alternative, enabling improved adhesion and coating durability through controlled surface chemistry and topography. Excessive hydrogel swelling after coating further compromises interfacial stability and mechanical performance, emphasizing the need for precise swelling control via crosslinking density modulation or composite formulations.

Formulation challenges also persist, particularly regarding the incorporation of hydrophobic drugs into hydrophilic hydrogel matrices. Limited solubility and poor dispersion can restrict drug loading and release control. Further, sterilization remains another critical obstacle. Conventional methods involving heat, radiation, or chemical exposure can disrupt hydrogel network integrity. Thus, shrinkage, dehydration, or crosslinking damage can occur, highlighting the need for sterilization protocols tailored specifically to hydrogel-coated implants.

Emerging high-throughput screening platforms also offer opportunities for the rapid identification of material formulations. These can be tailored for personalized implants, where patient-specific data guide the customization of mechanical and biological responses to meet individual therapeutic needs. In parallel, next-generation hydrogel technologies should explore their potential as biomedical interfaces in implantable bioelectronic devices for diagnostics, therapy, and tissue regeneration. Because of their hydrophilicity, ECM-mimicking architecture, and inherent biocompatibility, hydrogels can function as effective mediators between electrochemical components and living tissues. This hybrid interface enhances mechanical compliance and signal transduction while reducing immune response and interfacial mismatch. Collectively, such advances will facilitate the creation of bioelectronic systems that seamlessly integrate with biological environments.

Looking ahead, further progress in IHGs will depend on the integration of smart materials, advanced fabrication technologies, artificial intelligence, and bioelectronic interfaces. Stimuli-responsive hydrogel coatings that respond to physiological cues, such as pH, temperature, or inflammation, may enable controlled antimicrobial release or adaptive structural behavior. In parallel, 3D and 4D printing technologies offer precise spatial control and dynamic functionality, supporting patient-specific and multi-material designs. AI-driven approaches, including machine learning and computational modeling, provide tools for optimizing hydrogel properties related to physiological behavior, bioactivity, and drug release. Machine learning algorithms, molecular dynamics simulations, and computational modelling can be used to analyze large compositional and performance datasets. These tools enable the prediction, screening, and optimization of hydrogel properties, such as crosslinking density, swelling ratio, mechanical strength, and degradation kinetics, in relation to specific physiological environments. Future research should prioritize the integration of artificial intelligence (AI) and data-driven design frameworks to accelerate the development of implantable hydrogel systems. Finally, owing to their hydrophilicity, ECM-mimicking properties, biocompatibility, and mechanical compliance, hydrogels hold promise as effective interfaces for implantable bioelectronic systems in diagnostics, therapeutics, and tissue regeneration.

## CRediT authorship contribution statement

**Bruna E. Nagay:** Writing – original draft. **Leila Mamizadeh Janghour:** Writing – original draft. **Labiba K. El-Khordagui:** Writing – original draft, Writing – review & editing. **Behnam Akhavan:** Writing – original draft. **Valentim A.R. Barão:** Writing – original draft, Writing – review & editing. **Vimukthi Dananjaya:** Writing – original draft. **Chamil Abeykoon:** Writing – original draft. **Salma E. El-Habashy:** Writing – original draft. **Jagan Mohan Dodda:** Conceptualization, Writing – original draft, Writing – review & editing.

## Declaration of competing interest

The authors declare that they have no known competing financial interests or personal relationships that could have appeared to influence the work reported in this paper.

## Data Availability

The data described in the article are available at https://zenodo.org/records/18384878. We would appreciate if other researchers could benefit from our literature and results. This will foster discussions and collaboration among scientists worldwide.
